# Economic Design of a Novel Magnetic ZnO-Doped Biocomposite: An Integrated Advanced Ionic Theory and Statistical Physics Approach for Cr(VI) and Hg(II) Remediation

**DOI:** 10.3390/nano16090521

**Published:** 2026-04-25

**Authors:** Ahmed A. Bhran, Abdelrahman G. Gadallah, Raid Alrowais, Ahmed S. Aadli, Ahmed S. Elshimy

**Affiliations:** 1 Chemical Engineering Department, College of Engineering, Imam Mohammad Ibn Saud Islamic University (IMSIU), Riyadh 11432, Saudi Arabia; agadallah@imamu.edu.sa; 2 Department of Civil Engineering, College of Engineering, Jouf University, Sakakah 72388, Saudi Arabia; 3 Chemistry Labs. R&D, Aluminum Company of Egypt, Nag Hammadi 83642, Egypt; ahmed.s.aadli@sci.svu.edu.eg; 4 Faculty of Earth Science, Beni–Suef University, Beni Suef 62511, Egypt; ahmed.salah2407@esc.bsu.edu.eg

**Keywords:** hydrophobic scleroprotein discards, Cr(VI), Hg(II), advanced physics modeling, leachability analysis

## Abstract

A previously unexplored magnetic biocomposite (CMC-HSDs/Fe_3_O_4_) was developed through the valorization of hydrophobic scleroprotein discards (HSDs). The synthesized material was evaluated for its efficacy in the adsorption of Cr(VI) and Hg(II) ions from contaminated aqueous systems. The physicochemical properties of the synthesized CMC-HSDs/Fe_3_O_4_ nanocomposite were characterized using XRD, FTIR, BET, TG/DTG, FESEM, EDX, and elemental mapping. Subsequently, a Box–Behnken experimental design was employed to model and optimize the adsorption process for Cr(VI) and Hg(II), focusing on the critical parameters of solution pH, adsorbent dosage, and interaction time. Kinetic data were best fitted to the pseudo-first-order (PFO) model. Equilibrium isotherm analysis revealed that Cr(VI) adsorption followed the Langmuir model, while Hg(II) adsorption was better fitted by the Freundlich model. Advanced ionic calculations elucidated a consistent multimolecular adsorption mechanism for both ions, characterized by temperature invariance and a preferential vertical geometry of the adsorbed species. Through a production cost of 25.56 USD/kg, the biosorbent demonstrates excellent reusability, retaining 88.60% efficiency for Cr(VI) and 85.69% for Hg(II) after five adsorption–desorption cycles. Based on a 50 mg/L influent concentration, projected treatment costs are ~$3.50/100 L for Cr(VI) and ~$1.22/100 L for Hg(II), underscoring the nanocomposite’s economic feasibility for industrial deployment in advanced tertiary wastewater remediation.

## 1. Introduction

Intensified industrial and urban development acts as a primary driver of environmental contamination, particularly through the release of toxic heavy metals like chromium (Cr) and mercury (Hg) [1,2]. These hazardous substances are prevalent in waste streams from diverse industrial processes, including mining, smelting, and manufacturing [3]. The development of effective water purification methodologies is imperative due to the severe toxicological profile of heavy metals. Chronic exposure to these ions is a known etiology for numerous pathologies, ranging from central nervous system damage and gastrointestinal distress to malignant growth and chronic hypertension [4]. Research efforts are predominantly directed toward the creation of remediation technologies capable of diminishing heavy metal content in effluent to within legally mandated thresholds.

A spectrum of methodologies exists for the remediation of heavy metals, including chemical precipitation [5,6], ion exchange [7,8], and filtration [9,10]. Among these, adsorption is distinguished for its operational simplicity, rapid kinetics, and high efficacy, establishing it as a preeminent treatment technology [11,12]. Contemporary research initiatives are increasingly directed toward engineering novel sorbents with enhanced performance attributes. A significant advancement involves synthesizing sustainable composite materials from animal-derived biomass through hydrothermal carbonization (HTC), representing a promising trajectory in adsorbent design [13,14]. Hydrophobic scleroprotein discards (HSDs) have emerged as an efficient biomass-derived sorbent for pollutant sequestration, a utility derived from their favorable surface characteristics and status as a plentiful animal-derived byproduct. The material is predominantly keratin, a robust fibrous protein, making it a globally relevant repository of organic polymers. The material’s architecture is distinguished by a significant density of functional groups, including amine (–NH_2_), carboxyl (–COOH), and hydroxyl (–OH) moieties. These active sites are instrumental in facilitating the effective sequestration of contaminants, demonstrating particular efficacy for the extraction of heavy metals and organic pollutants from aqueous environments [15,16,17]. The inherent porosity and surface area of these materials significantly boost their adsorption efficiency, while their hydrophobic nature facilitates the targeted extraction of non-polar contaminants. In contrast to traditional adsorbents, carbonized HSDs exhibit notable benefits, such as cost-effectiveness and sustainable sourcing, making it highly compatible with circular economic models [16]. The upcycling of HSDs into adsorbent media offers a twofold environmental benefit: it provides a renewable resource for the sequestration of hazardous contaminants while also valorizing animal waste. Its binding capacity, which is markedly improved by structural modifications such as cross-linking, positions it as an eco-friendly replacement for hazardous industrially produced alternatives.

The suitability of cellulose as a versatile raw material stems from its natural abundance and low cost. Structurally defined by β-1,4 glycosidic linkages between d-glucose units, it possesses a chemistry amenable to various derivatizations. These chemical alterations can engineer specific characteristics like water solubility, significantly expanding its potential uses [18]. This research employs carboxymethyl cellulose (CMC), a hydro-soluble derivative, as a model compound. A standard synthetic approach for fabricating cellulose-based adsorbents involves the graft copolymerization of hydrophilic monomers with a crosslinking agent, resulting in a hydrogel sorbent matrix [16]. The pronounced efficacy of these materials in sequestering heavy metal ions and organic dyes from aqueous media is attributable to several distinct physicochemical characteristics. These include a versatile polymer framework amenable to functionalization with diverse chelating agents, a permeable internal architecture, and an extensive specific surface area.

Although carboxymethyl cellulose (CMC) has been explored individually for heavy metal adsorption, most previous studies have employed CMC either in isolation or as a simple hydrogel. For example, Zhang et al. (2025) fabricated carboxymethyl cellulose/pectin-based hydrogel beads that achieved adsorption capacities of 139.4 mg/g for Cd^2+^, 270.3 mg/g for Pb^2+^, and 143.6 mg/g for Cu^2+^ [19]. Similarly, Rahman et al. (2026) utilized deacetylated chitin-modified, biogenic waste-derived Fe_3_O_4_ nanoparticles for Mn(VII) removal, reporting a capacity of approximately 450.18 mg/g [20]. Despite these advances, no prior work has synergistically integrated CMC with valorized hydrophobic scleroprotein discards (HSDs) and magnetic Fe_3_O_4_ nanoparticles into a single biocomposite. This ternary assembly uniquely harnesses the complementary advantages of all three components: the high density of functional groups from HSDs, the hydrophilic porous matrix from CMC, and the magnetic recoverability along with Lewis acid sites from Fe_3_O_4_. This unprecedented integration yields superior adsorption capacities—236.7 mg/g for Cr(VI) and 162.07 mg/g for Hg(II).

The integration of magnetic nanoparticles (Fe_3_O_4_) into various matrices facilitates the fabrication of composite materials with tailored functionalities. Consequently, cellulose-based composites incorporating Fe_3_O_4_ have garnered significant scientific interest, as they exhibit synergistic properties that merge the distinct advantages of both constituents, such as biocompatibility and inherent biodegradability [21]. Furthermore, the magnetic Fe_3_O_4_ nanoparticles were specifically chosen for three primary reasons: (i) they enable rapid and facile separation of the spent adsorbent from treated water using an external magnetic field, eliminating the need for energy-intensive centrifugation or filtration steps; (ii) they provide Lewis acid sites (Fe centers) that can coordinate with heavy metal ions, thereby enhancing adsorption capacity; and (iii) they improve the mechanical and thermal stability of the composite, as evidenced by the TG/DTG analysis (Section 3.1.4). Unlike non-magnetic adsorbents that require complex recovery procedures, the magnetic property of CMC-HSDs/Fe_3_O_4_ significantly reduces operational costs and time in large-scale water treatment applications [22].

Guided by preceding evidence, this investigation pioneers a synergistic methodology for fabricating advanced nanocomposite adsorbents. The design is predicated on the deliberate assembly of three key components: tailored magnetic nanoparticles, repurposed avian keratin biomass, and a porous cellulose hydrogel matrix. The sequestration of heavy metals in this system is primarily facilitated by electrostatic interactions, wherein the cationic or anionic surface of the magnetic cellulose nanocomposite attracts species such as Cr(VI) and Hg(II) complexes. Furthermore, the composite’s intrinsic magnetism enables its straightforward retrieval from solution post-adsorption using an external magnetic field.

This study introduces a novel, eco-friendly biosorbent synthesized by integrating valorized hydrophobic scleroprotein discards (HSDs) with carboxymethyl cellulose (CMC) and magnetite nanoparticles (Fe_3_O_4_) form a CMC-HSDs/Fe_3_O_4_ biocomposite. The material fulfills a key research need by combining these components for the first time, demonstrating exceptional adsorption capacity for both Cr(VI) and Hg(II) ions. A comprehensive suite of analytical techniques, including FTIR, XRD, TG/DTG, BET surface area analysis, FESEM, EDX, and elemental mapping, was employed to characterize its structural and surface properties, confirming its potential as a significant innovation in wastewater remediation. Optimal adsorption conditions were subsequently established through systematic optimization using a Box–Behnken experimental design. The adsorption kinetics and equilibrium isotherms for Cr(VI) and Hg(II) were analyzed using classical models. To elucidate the molecular-scale adsorption mechanism, advanced statistical physics modeling was employed, providing deeper insight into the binding configuration and energy of the ions on the CMC-HSDs/Fe_3_O_4_ active sites. Thermodynamic analysis, based on Gibbs free energy, internal energy, and entropy, revealed the nature and energy profile of the removal process, clarifying the governing interfacial mechanisms. Overall, this study pioneers an innovative synthesis route, integrating biopolymers, metallic nanoparticles, and biogenic solid waste to fabricate a high-performance CMC-HSDs/Fe_3_O_4_ nanosorbent.

## 2. Materials and Methods

### 2.1. Materials and Reactants

The principal adsorbent material consisted of hydrophobic scleroprotein discards sourced from duck feathers, which were collected from domestic sources. The chemicals used in this investigation, including carboxymethyl cellulose, potassium chromate, iron(II) sulfate heptahydrate, ammonium hydroxide (25%), and iron(III) chloride hexahydrate, were obtained from Advent Chembio Pvt. Ltd. (Mumbai, India). Mercuric sulfate was sourced from Merck, located in Darmstadt, Germanythe. The pH of all experimental solutions was adjusted to the desired values using sodium hydroxide or hydrochloric acid.

### 2.2. Synthesis of CMC-HSDs/Fe_3_O_4_

**Figure 1:**
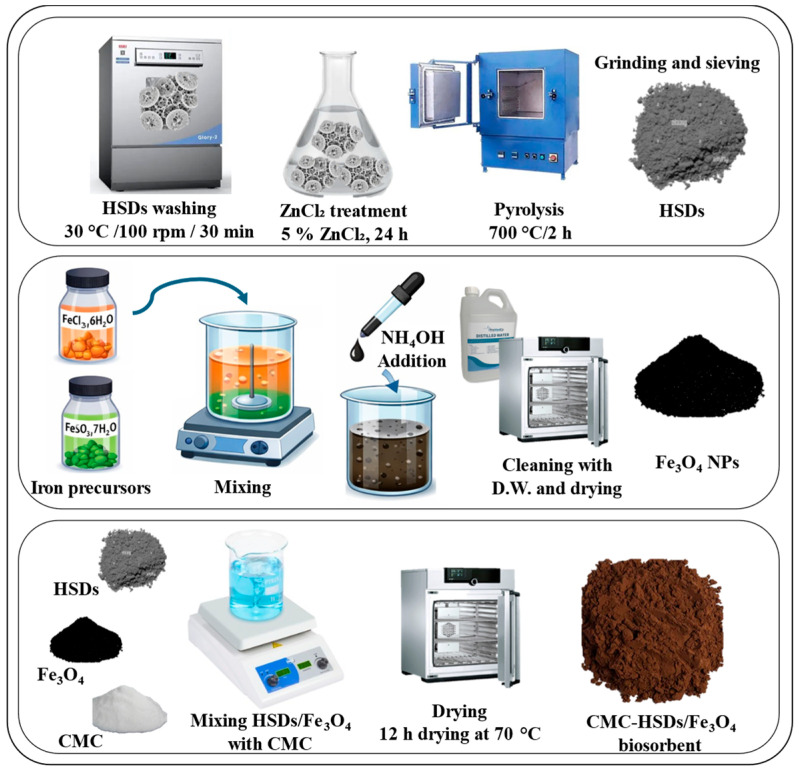
Schematic of the key synthesis steps for CMC-HSDs/Fe_3_O_4_.

The CMC-HSDs/Fe_3_O_4_ biocomposite was synthesized through a sequential methodology outlined in Figure 1. The initial stage involved processing a predetermined mass of HSDs through a comprehensive aqueous cleansing cycle in an industrial washing machine at 30 °C and 100 rpm for 30 min, maintaining detergent-free conditions throughout the procedure. The purified feathers subsequently underwent chemical activation through 24 h immersion in a 5% zinc chloride (ZnCl_2_) solution. This pretreatment was followed by pyrolysis in a calcination furnace at 700 °C for 2 h. The resulting activated carbon material was then mechanically pulverized and sieved to isolate particles smaller than 75 μm for further application. Secondly, a co-precipitation protocol was utilized for Fe_3_O_4_ nanoparticle fabrication, achieved by combining iron(III) chloride hexahydrate and iron(II) sulfate heptahydrate in ammonium hydroxide solution to facilitate nucleation and growth [23]. In the final synthesis step, 10.0 g of HSDs was homogenized with 2.0 g of Fe3O4 at 100 rpm. This composite was subsequently stabilized using a binding matrix of carboxymethyl cellulose (CMC), prepared by dissolving 2.0 g of CMC powder in 100 mL of distilled water to yield a 2.0 wt.% aqueous solution. This polymeric solution was incorporated into the binary HSDs/Fe_3_O_4_ adsorbent and agitated for 60 min at 50 °C. Following this integration, the mixture was converted into a solid phase through 12 h of drying at 70 °C, with the final material ground and size-classified to sub-80 µm particles.

### 2.3. Characterization of the Synthesized CMC-HSDs/Fe_3_O_4_ Nanocomposite

The crystalline phases present in both the raw HSDs and the synthesized CMC-HSDs/Fe_3_O_4_ composite were characterized by X-ray diffraction (XRD) analysis. Measurements were performed on a PANalytical Empyrean diffractometer using Cu-Kα radiation (λ = 0.154 nm), with diffraction patterns collected across a 2θ range of 10–80° at operating conditions of 40 kV and 30 mA. Chemical functional groups inherent to the HSDs and the resulting adsorbent were characterized by FTIR spectroscopy. Measurements conducted on a Bruker Optics VERTEX 70 instrument captured mid-infrared spectra (400–4000 cm^−1^), enabling identification of molecular vibrations diagnostic of specific chemical moieties. The thermal stability and decomposition kinetics of the CMC-HSDs/Fe_3_O_4_ composite were evaluated in a nitrogen atmosphere using thermogravimetric analysis. Experiments performed on a stream thermogravimetric (TG) analyzer involved heating specimens from 50 to 778 °C at 10 °C min^−1^ while maintaining a 50 mL min^−1^ nitrogen flow, with simultaneous recording of mass loss and its derivative (DTG). The surface morphology of the CMC-HSDs/Fe_3_O_4_ biocomposite was characterized using a Thermo Fisher Scientific Quanta FEG 250 field emission scanning electron microscope (FESEM). Complementary elemental composition and distribution were ascertained through energy-dispersive X-ray (EDX) spectroscopy, while textural properties including specific surface area and pore structure were quantified from nitrogen physisorption isotherms using Brunauer–Emmett–Teller (BET) theory on a Micromeritics TriStar II 3020 system.

### 2.4. Cr(VI) and Hg(II) Adsorption Studies

The sequestration capacity of the CMC-HSDs/Fe_3_O_4_ composite for Cr(VI) and Hg(II) ions was methodically examined through batch adsorption studies. The critical process variables were rigorously optimized to quantify their individual and combined effects on removal performance. These variables included solution pH (2–9), adsorbent mass (5–30 mg), interaction time (5–150 min), and initial metal concentration (10–160 mg/L). All adsorption experiments were performed in triplicate using 50 mL centrifuge tubes under standardized conditions. Particular emphasis was placed on evaluating pH effects across acidic to moderately alkaline ranges to elucidate its role in adsorption mechanisms. Isotherm studies were performed using Cr(VI) and Hg(II) solutions spanning concentrations of 10–160 mg/L, with thermal regulation maintained at 25, 40, and 55 °C throughout the experimental series. Time-dependent adsorption profiles were generated from 100 mg/L metal solutions sampled between 5 and 150 min under constant orbital shaking at 200 rpm to maintain uniform mass transfer conditions. Following the adsorption process, the remaining concentrations of Cr(VI) and Hg(II) in the centrifuged supernatants were determined through spectrophotometric measurement. The metal uptake at equilibrium (*qₑ*) and at specific time intervals (*qₜ*) were computed employing Equations (1) and (2), respectively.

(1) $$q_{e}=(C_{O}-C_{e})\frac{V}{m}$$

(2) $$q_{t}=(C_{O}-C_{t})\frac{V}{m}$$

The experimental notation defines C_0_ as the initial concentration (mg/L) of Cr(VI) and Hg(II) ions, with C_e_ representing their respective concentrations at adsorption equilibrium. The parameter C_t_ quantifies the residual metal concentration at time t, while V and m designate the solution volume (L) and mass (g) of the CMC-HSDs/Fe_3_O_4_ adsorbent, respectively. 

### 2.5. Modeling of Heavy Metals Adsorption by CMC-HSDs/Fe_3_O_4_

Understanding molecular-scale adsorption processes demands rigorous quantification of key physicochemical descriptors contained within equilibrium modeling formalisms. The Langmuir and Freundlich models constitute fundamental theoretical frameworks in interfacial science, valued for their consistent application in quantifying adsorption phenomena under varied experimental regimes [24,25]. The comprehensive investigation of adsorption phenomena across varying dimensional scales requires implementation of sophisticated theoretical approaches founded in statistical physics principles [26,27]. The convergence of experimental data with theoretical formalisms facilitates a multi-scale interpretation of adsorption behavior. Full procedural specifications for equilibrium, kinetic, and thermodynamic analysis are documented in the Supplementary Material.

### 2.6. Experimental Optimization Design

Process optimization was conducted using Response Surface Methodology (RSM), a statistical framework for modeling variable-response relationships. This methodology reduces experimental requirements while accurately capturing parameter interactions and system performance. A Box–Behnken Design (BBD) incorporating quadratic modeling was implemented to thoroughly investigate both individual parameter effects and their synergistic interactions. The Design-Expert software (Version 12.0) used for experimental design, statistical analysis, and process optimization was sourced from Stat-Ease Inc., headquartered in Minneapolis, Minnesota, USA. This systematic investigation quantified and optimized three critical operational parameters governing Cr(VI) and Hg(II) removal: solution pH, adsorbent mass, and contact time. Process optimization employed a BBD framework to systematically investigate adsorption efficiency. Table S1 details the coded parameter levels implemented within the experimental design matrix. The resulting response data were analyzed using a second-order polynomial model (Equation (3) to predict the sequestration efficiency of Cr(VI) and Hg(II) contaminants [28,29].

(3) Y=βo+∑βi Xi+∑βii Xi2+∑∑βij Xi Xj

Within this mathematical framework, Y signifies the sequestration efficiency for Cr(VI) and Hg(II) ions, whereas Xi and Xj correspond to coded independent variables. The model coefficients βi, βii, and βij quantify linear, quadratic, and interactive contributions, respectively, with βo defining the constant term. Each experimental condition involved introducing a specified adsorbent mass (Table S2) into 25 mL of metal ion solution under continuous agitation. The resulting contaminant removal percentage (R, %) was subsequently calculated using Equation (4) [29,30].

(4) $$R\;(\%)=\frac{C_{O}-C_{e}}{C_{O}}\times 100$$

The sequestration efficiency (R, %) for Cr(VI) and Hg(II) ions was calculated from their initial (C_0_) and equilibrium (C_e_) concentrations. Table S2 comprehensively presents the experimental and predicted results obtained from all seventeen trials constituting the BBD matrix.

### 2.7. Desorption, Leachability, and Environmental Stability Assessment of the CMC-HSDs/Fe_3_O_4_

To assess the reusability of the CMC-HSDs/Fe_3_O_4_ nanocomposite, a sample saturated with Cr(VI) and Hg(II) ions under optimal adsorption conditions was recovered and subjected to a systematic desorption study. This procedure was performed at a regulated temperature of 25 °C. Using 0.1 M HCl as the eluent with a solid-to-liquid ratio of 1 g/L, the mixture was agitated continuously for 60 min. The resulting desorption efficiency was subsequently calculated based on the measured concentrations of eluted Cr(VI) and Hg(II) ions. To ascertain the adsorbent’s stability for repeated application, five sequential adsorption–desorption cycles were executed. Each cycle comprised an adsorption phase at pH 2 for Cr(VI) and pH 5 for Hg(II), succeeded by material regeneration with 0.1 M HCl. Following every desorption step, the regenerated biocomposite was washed thoroughly with deionized water to eliminate any residual acid. It was then dried in an oven at 80 °C for 6 h in preparation for its reuse in the next adsorption cycle. The contaminant removal efficiency was measured with precision after every regeneration cycle using standard analytical techniques. This quantification confirmed the biocomposite’s capacity to maintain its high-performance adsorption capabilities through repeated applications.

In addition to standard characterization, the material containing immobilized Cr(VI) and Hg(II) was also tested for its leaching potential under two rigorous scenarios designed to simulate plausible real-world disposal or in situ application environments. Of the two simulated environmental scenarios, the first, termed Acidic Leachability, immersed the composite in a pH 4 buffer solution for 72 h under constant mild agitation. The second, designated Ionic Strength Leaching, subjected the material to a 0.1 M sodium chloride solution for the same period, replicating the ionic strength commonly found in saline groundwater. During both leaching experiments, supernatant samples were collected at predetermined intervals. Their concentrations of Zn, Fe, Cr(VI), and Hg(II) were subsequently determined by atomic absorption spectroscopy to measure any component release.

## 3. Results and Discussions

### 3.1. Characterization of Fabricated CMC-HSDs/Fe_3_O_4_ Biosorbent

#### 3.1.1. FTIR Spectroscopy

**Figure 2:**
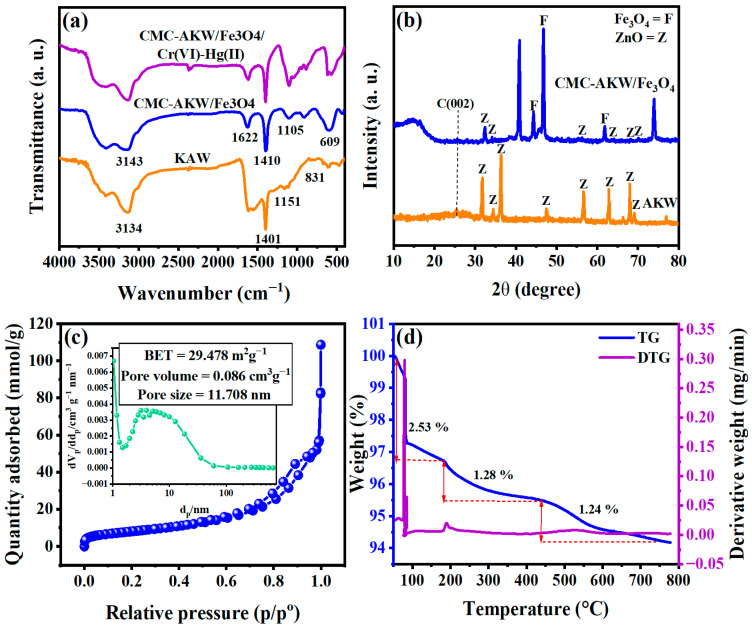
(**a**) FTIR spectrum of HSDs, CMC-HSDs/Fe_3_O_4_, and CMC-HSDs/Fe_3_O_4_-Cr(VI)/Hg(II), and (**b**) XRD of HSDs and CMC-HSDs/Fe_3_O_4_ where (**c**) BET analyses and (**d**) TGA studies of CMC-HSDs/Fe_3_O_4_ biocomposite.

To analyze the changes in surface functional groups after composite formation, FTIR spectroscopy was employed. Figure 2a displays the comparative spectra for HSDs, the CMC-HSDs/Fe_3_O_4_ biosorbent, and the material following the adsorption of Cr(VI) and Hg(II), covering the range from 400 to 4000 cm^−1^. In the FTIR analysis of HSDs, keratin’s distinctive molecular fingerprint is evident. Principal spectral features correspond to the amide (-CO-NH-) linkage, accompanied by identifiable stretches attributable to -NH_2_, -C=NH, and -C-H functional groups [31]. The broad absorption band centered near 3134 cm^−1^ corresponds to superimposed stretching modes arising from hydrogen-bonded amide A (-N-H) groups and adsorbed water (-O-H) molecules. Additionally, a vibrational mode at 1401 cm^−1^ is assigned to the scissoring deformation of -CH_2_- groups. Furthermore, the infrared spectrum displayed characteristic bands at 1151 and 831 cm^−1^, attributable to the stretching mode of the carbon-nitrogen (-C-N-) bond. Moreover, the concurrent identification of all key absorptions, those specific to the N-H, C=O, and C-N bonds as well as the characteristic CNH functional group, constitutes conclusive verification for the presence of amino acids. The elemental monomers establish the peptide bonds required for keratin’s supramolecular organization [32]. In addition to preserving the fingerprint regions of HSDs, the composite biosorbent exhibits a defining spectral feature at 609 cm^−1^, which is characteristic of Fe–O bond oscillations. This result definitively confirms the incorporation of Fe_3_O_4_ nanoparticles, thereby imparting magnetic functionality to the synthesized nanosorbent material [33]. Upon examining the FTIR spectra of the CMC-HSDs/Fe_3_O_4_ composite illustrated in Figure 2a, a distinct and prominent absorption band is evident at 3143 cm^−1^. This feature can be ascribed to the presence of hydroxyl functional groups originating from the carboxymethyl cellulose component [34]. Furthermore, the distinct peaks identified at 1622 cm^−1^ and 1410 cm^−1^ are characteristic of the asymmetric and symmetric stretching vibrations of the carboxylate anion (COO^−^) [35]. The presence of a six-membered cyclic ether structure is confirmed by an absorption band at 1105.25 cm^−1^, which arises from the asymmetric stretching vibration of the C–O–C linkage [36]. The observed results offer conclusive verification that the CMC-HSDs/Fe_3_O_4_ nanosorbent has been successfully synthesized. After the sequestration of Cr(VI) and Hg(II), the FTIR spectra underwent significant alteration. This was evident in both the variation in intensity and the displacement in wavenumber for characteristic vibrational bands, specifically those corresponding to O–H, C–O, Fe–O, and C=C functional moieties. Such alterations in the spectroscopic data corroborate the proposed adsorption mechanism, thereby highlighting the essential contribution of the identified surface functional groups to the sequestration of both Cr(VI) and Hg(II).

#### 3.1.2. Mineralogical Investigations

Crystallographic verification for both the HSDs precursor and the resulting CMC-HSDs/Fe_3_O_4_ biocomposite is provided by the XRD patterns displayed in Figure 2b, which cover a 2θ scan from 10 to 80 degrees. A sequence of distinct and prominent diffraction peaks defines the XRD profile of the HSD material. These peaks, observed at 2θ values of approximately 31.78°, 34.51°, 36.42°, 47.39°, 56.91°, 62.74°, 67.87°, and 69.24°, are systematically indexed to the (100), (002), (101), (102), (110), (103), (200), and (112) crystal planes. This pattern is indicative of a predominantly hexagonal wurtzite structure for ZnO. The development of highly crystalline zinc oxide phases is confirmed by the sharp diffraction peaks [37]. This crystalline structure was achieved through the thermal treatment (calcination) of a keratin precursor loaded with zinc chloride. Subsequent examination of the diffraction pattern identifies a low-intensity, diffuse feature centered at approximately 25.18° 2θ. This signal is attributed to residual amorphous carbonaceous material originating from the thermal degradation of the organic keratin matrix. Critically, the lack of any additional diffraction maxima verifies the phase purity of the synthesized zinc oxide. The XRD pattern of the CMC-HSDs/Fe_3_O_4_ composite, while showing the principal crystalline phases of HSDs, revealed decreased ZnO peak intensities. This change signifies a structural reorganization within the material framework upon functionalization. Additionally, the XRD pattern of the nanocomposite features distinct reflections at 20 angles of 46.84° and 61.76°, which are indexed to specific crystallographic planes inherent to magnetite (Fe_3_O_4_) [26,38]. The incorporation of Fe_3_O_4_ nanoparticles not only imparts magnetic functionality but also enhances adsorption through surface coordination. The iron atoms on the Fe_3_O_4_ surface act as Lewis acid sites, enabling coordination with electron-rich functional groups such as –NH_2_, –COOH, and –OH present in HSDs and CMC. This coordination increases the density of active binding sites and promotes electrostatic interactions with Cr(VI) and Hg(II) ions. Additionally, the Fe–O bonds, confirmed by the FTIR peak at 609 cm^−1^, participate in surface complexation mechanisms. This definitive phase identification confirms the successful incorporation and structural integrity of the magnetic nanoparticles within the synthesized biocomposite matrix [26].

#### 3.1.3. BET Studies and Surface Characteristics

**Figure 3:**
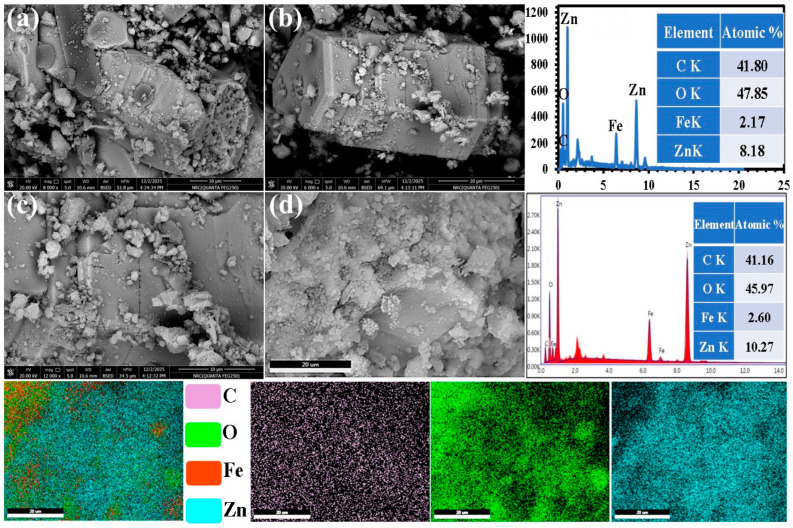
FESEM imaging (**a**–**c**) and EDX spectroscopy with elemental mapping (**d**) characterized the microstructure, morphology, and composition of the CMC-HSDs/Fe_3_O_4_ biosorbent.

The textural properties of the synthesized CMC-HSDs/Fe_3_O_4_ nanosorbent were characterized by applying the BET method. This analysis produced a specific surface area of 29.478 m^2^/g, an average pore diameter of 11.7 nm, and a corresponding pore volume of 0.086 cm^3^/g. Beyond merely possessing a significant surface area, the nanocomposite’s effectiveness is fundamentally driven by a synergistic interplay between its available surface area and superior chemical functionality. This functionality, derived from its ternary material composition (i.e., CMC, HSDs, and Fe_3_O_4_), provides multifunctional binding sites for chemical bonding and a native capacity for electrostatic adsorption. Moreover, the characterization of the CMC-HSDs/Fe_3_O_4_ composite revealed a pore size distribution below 50 nm, as depicted in Figure 2c. This measurement aligns with the established classification for mesoporous materials, thereby confirming the mesoporous nature of the synthesized composite [39]. The nanocomposite’s nitrogen adsorption–desorption isotherm (Figure 3c) exhibits a Type IV physisorption profile accompanied by an H_4_ hysteresis loop, as classified within the IUPAC framework [28]. This form of isotherm is characteristic of mesoporous adsorbent materials, which display limited energetic interaction with adsorbed species. This distinct porous framework contributes to a high sequestration capacity through two key functions: the provision of active sites for interfacial binding and the guarantee of unrestricted intraparticle diffusion. A notable concordance exists between these findings and the FESEM analysis (Figure 3), indicating a material architecture defined by porosity and the agglomeration of irregular particulate forms. This specific structural configuration is inherently beneficial for sequestration applications, as the interconnected pore matrix delivers both an extensive reactive surface area and an intrinsic capacity for structural adaptation, collectively contributing to enhanced functional efficacy.

Regarding the controllability of pore structure, the number and size of pores in the CMC-HSDs/Fe_3_O_4_ composite can be tuned by adjusting several synthesis parameters. Specifically: (i) the ZnCl_2_ activation concentration (tested at 5% in this study) influences the degree of porosity; higher concentrations generally increase pore volume but may compromise structural integrity; (ii) the pyrolysis temperature (700 °C in this work) determines the extent of carbonization and pore development; (iii) the CMC-to-HSD mass ratio affects the hydrogel network density and subsequent pore architecture; and (iv) the incorporation of Fe_3_O_4_ nanoparticles introduces additional interstitial spaces at the nanoparticle-polymer interface. Systematic optimization of these parameters could yield composites with tailored pore sizes, offering flexibility for targeting specific contaminants based on their molecular dimensions. This tunability represents a key advantage of the ternary composite design.

#### 3.1.4. High-Temperature Stability Assessment

The thermal degradation profile, obtained via TG/DTG analysis from 50 to 778 °C (Figure 2d), demonstrates the material’s robust carbonaceous character. The TGA curve indicates minimal decomposition, with a cumulative weight loss of merely 5.05% recorded at 774 °C, yielding a substantial residual fraction of 94.95%. These profiles elucidate the decomposition properties of the CMC-HSDs/Fe_3_O_4_ biosorbent. The TGA profile exhibited three discrete stages of mass reduction: initial moisture release at temperatures below 200 °C, followed by the decomposition of hemicellulose and cellulose occurring between 200 and 400 °C, concluding with the complete thermal degradation of cellulose constituents above 400 °C. The progression of thermal decomposition significantly influenced the overall yield of the biosorbent composite, as evidenced by successive reductions in mass. Following a loss of 2.53% up to 180.12 °C, the mass diminished by a further 1.28% upon reaching 437.90 °C, and ultimately by an additional 1.24% when heated to 738.99 °C (Figure 2d). The observed sequential degradation culminates in enhanced thermal stability at 700 °C, as indicated by a larger residual mass compared to the CMC-HSDs/Fe_3_O_4_ composite. This greater residue points to an improved stability threshold and, consequently, a higher resistance to structural breakdown under thermal stress.

#### 3.1.5. Morphological Characteristics of CMC-HSDs/Fe_3_O_4_ Biocomposite

Analysis of the morphological characteristics and elemental spatial arrangement within the CMC-HSDs/Fe_3_O_4_ biocomposite was performed using FESEM/EDX and elemental mapping. The FESEM results confirm the effective synthesis of a tri-phase, heterogeneous adsorbent material, as illustrated in Figure 3a–d. The microstructure exhibits marked morphological variation, presenting two clearly distinguishable forms: developed ZnO crystals that serve as a substrate for deposited CMC particles and a porous architecture embedded within a fragmented HSD matrix (Figure 3a). This structural diversity implies the occurrence of distinct formation pathways, probably influenced by localized differences in precursor interactions or energy conditions during the fabrication process. The nanocomposite matrix features interstitial regions and inherent voids that collectively generate a significant porous network. This pore structure is essential for enabling effective mass transport, which significantly enhances the material’s performance in the removal of Cr(VI) and Hg(II). Moreover, the holocrystalline shape detected in Figure 3b is attributed to the hexagonal wurtzite structure as proved by XRD data in Figure 2b. After the calcination of ZnCl_2_-activated HSDs, distinct crystalline domains were characterized as the wurtzite phase of ZnO was formed. These developed ZnO crystals function as a structural foundation for the subsequent integration of carbonized keratin phases and CMC particles. The identification of Fe_3_O_4_ nanoparticles within the composite matrix is presented in Figure 3c. These nanoparticles, noted for their diminutive size and high reflectivity, are disseminated in the CMC-HSDs/Fe_3_O_4_ material.

EDX spectroscopic quantification (Figure 3d) established the elemental profile of the biosorbent, which is dominated by carbon (41.16%) and oxygen (45.97%), with significant quantities of zinc (10.27%) and iron (2.60%). This composition provides conclusive evidence for the successful synthesis of the target CMC-HSDs/Fe_3_O_4_ nanosorbent, confirming the incorporation of all precursor materials. Spatial analysis via elemental mapping further elucidated the composite’s microstructure. The significant distribution observed for carbon (purple), oxygen (green), and zinc (blue) signifies a high degree of dispersion. This microscale uniformity substantiates the quantitative compositional data, demonstrating that the integration of constituents was not only successful in bulk but also achieved a coherent and well-mixed micro-architecture.

### 3.2. The CMC-HSDs/Fe_3_O_4_ Evaluation for Cr(VI) and Hg(II) Adsorption

#### 3.2.1. Impact of Solution Acidity

**Figure 4:**
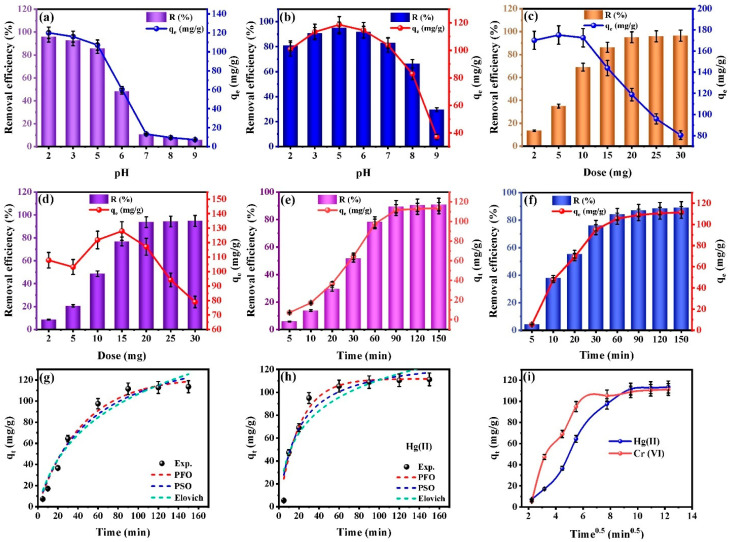
Effects of pH and dose on the uptake of Cr(VI) (**a**,**c**) and Hg(II) (**b**,**d**); kinetic studies of Cr(VI) and Hg(II) adsorption: effect of contact time (**e**,**f**), kinetic modeling (**g**,**h**), and LIPD modeling (**i**).

The solution acidity fundamentally regulates adsorption performance through concurrent modifications to both contaminant molecular speciation and adsorbent surface charge properties [40]. The pH drift method determined the point of zero charge (pH_pzc_) for the CMC-HSDs/Fe_3_O_4_ biocomposite to be 6.67 (Figure S1). The adsorbent surface carries a net positive charge in environments more acidic than this value, transitioning to a net negative charge under alkaline conditions. This fundamental alteration in surface electrochemistry directly regulates the pH-responsive sequestration behavior quantified during subsequent individual analysis of Cr(VI) and Hg(II) ion removal. Regarding the Cr (VI) ions, the analysis of the CMC-HSDs/Fe_3_O_4_ magnetic biocomposite revealed a significant pH-dependent characteristic in its adsorption performance. The uptake capacity for Cr (VI) diminished substantially from 120.03 mg·g^−1^ at pH 2 to 7.29 mg·g^−1^ at pH 9, culminating in a 90.2% reduction in removal efficiency across the tested pH range. The observed decline in adsorption with rising pH, presented in Figure 4a, originates from the surface charge characteristics of the composite. Under low-pH conditions, functional groups undergo protonation, creating a positively charged surface favorable for the electrostatic sequestration of anionic Cr (VI) species. The speciation of Cr (VI) is a critical factor governing its removal; under acidic conditions (pH < 4), the dominant CrO_4_^2−^ anion exhibits strong electrostatic affinity for the protonated, positively charged functional groups (e.g., -NH_3_^+^, -OH_2_^+^) on the adsorbent surface, thereby maximizing uptake [41,42,43]. Conversely, in alkaline environments (pH > 8.0), the CMC-HSDs/Fe_3_O_4_ composite develops a net negative surface charge. This electrostatic repulsion between the anionic contaminant species and the negatively charged surface sites actively impedes sequestration, directly explaining the diminished Cr(VI) removal efficacy observed under these conditions [44].

In contrast, the CMC-HSDs/Fe_3_O_4_ nanosorbent exhibited maximal Hg(II) ion sequestration at pH 5.0, achieving a removal efficiency of 94.92% (Figure 4b). A marked reduction in uptake performance was consistently observed across all other pH values tested. The superior adsorption performance under acidic conditions is mechanistically explained by the composite’s surface charge properties. As established by pH_ZPC_ measurements (Figure S1), the material maintains a positive charge within the pH range of 2–6, promoting a more favorable electrostatic interaction with anionic contaminants [45]. This enhanced removal is attributable to a greater degree of Hg(II) cation protonation, which intensifies the electrostatic forces toward the negatively charged hydroxyl moieties from physisorbed water on the CMC-HSDs/Fe_3_O_4_ nanocomposite surface—a mechanism established as the primary pathway for uptake [45]. The sequestration of Hg(II) could be governed by two principal factors: intraparticle diffusion into the adsorbent’s pore network and favorable electrostatic forces.

Regarding the oxidation state dependence, the CMC-HSDs/Fe_3_O_4_ composite was designed primarily for Hg(II) removal, which is the most common and toxic form of mercury in contaminated wastewater. Hg(0) (elemental mercury) is insoluble in water and is typically removed by different mechanisms (e.g., amalgamation or volatilization), while methylmercury (CH_3_Hg^+^) is an organometallic species with different coordination chemistry. Adsorption experiments focused exclusively on Hg(II) because it represents the predominant oxidation state in industrial effluents (e.g., from chlor-alkali plants, gold mining, and battery manufacturing) [46].

#### 3.2.2. Impact of CMC-HSDs/Fe_3_O_4_ Dosage on Cr(VI) and Hg(II) Adsorption Performance

A critical determinant of adsorption performance is the dosage of the CMC-HSDs/Fe_3_O_4_ biocomposite, which directly controls the number of accessible reactive sites. This parameter was rigorously investigated by testing a spectrum of adsorbent quantities between 2 and 30 mg. Figure 4c,d illustrate a distinct relationship for both Cr(VI) and Hg(II): while the adsorption capacity per unit mass exhibits an inverse trend with increasing adsorbent dosage, the overall removal efficiency demonstrates a concurrent positive trend. This divergence is attributed to the increased total number of active sites available for binding at higher adsorbent masses, which promotes greater total contaminant sequestration even as the specific capacity diminishes [47]. At fixed contaminant concentrations, increasing the adsorbent mass leads to a lower contaminant-to-site ratio, which suppresses the adsorption capacity (*qₑ*) at higher dosages. A systematic assessment of the CMC-HSDs/Fe_3_O_4_ performance established 20 mg as the optimal mass, and this quantity was therefore standardized for all subsequent experimental procedures.

### 3.3. Kinetic Modeling of Adsorption

Kinetic studies of adsorption are fundamental for assessing equilibrium attainment times and elucidating rate-controlling mechanisms [48]. The temporal adsorption profiles of Cr(VI) and Hg(II) on the CMC-HSDs/Fe_3_O_4_ biosorbent (Figure 4e,f) reveal their respective time-dependent uptake patterns. The adsorption kinetics for both Cr(VI) and Hg(II) can be segmented into three clear regimes: (i) an initial fast stage (t < 30 min) with near-instantaneous uptake; (ii) a transitional diffusion-controlled phase (30–90 min); and (iii) a final approach to equilibrium (90–150 min), during which adsorption rates steadily decrease until saturation. The high density of available active sites on the CMC-HSDs/Fe_3_O_4_ composite results in remarkably rapid initial adsorption kinetics, indicating strong binding affinity for both Cr(VI) and Hg(II) contaminants [49]. In the intermediate phase, kinetics shift to a diffusion-controlled regime dominated by intra-particle mass transfer, resulting in substantially slower contaminant uptake. At equilibrium (t ≥ 150 min), pollutant uptake reaches a steady state as surface sites become saturated and a dynamic adsorption–desorption equilibrium is established, making additional sequestration unfavorable.

**Figure 5:**
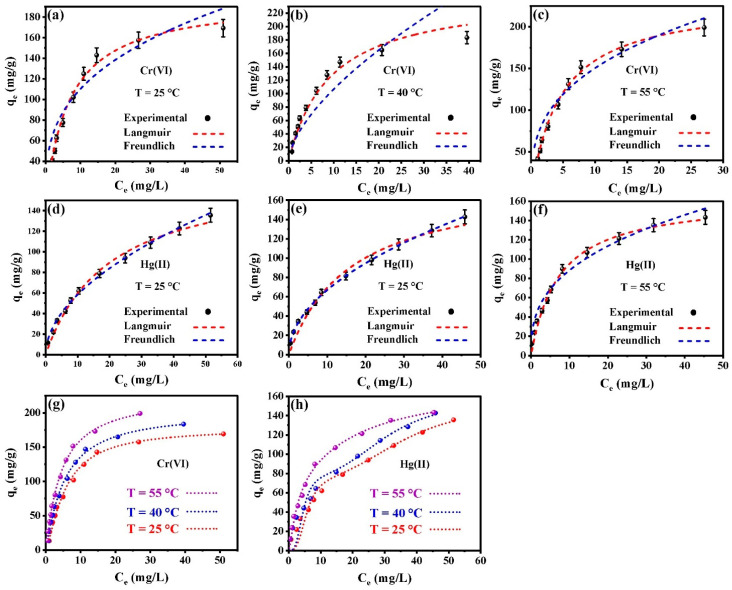
Comparison of nonlinear isotherm models (Langmuir, Freundlich) and advanced ionic calculations (monolayer, double-layer) for Cr(VI) (**a**–**c**,**g**) and Hg(II) (**d**–**f**,**h**) removal.

Nonlinear regression analysis of the *q_t_* versus *t* profiles for Cr(VI) and Hg(II) (Figure 4g,h) was used to derive the kinetic parameters corresponding to the Pseudo-First-Order (PFO), Pseudo-Second-Order (PSO), and Elovich models [50,51,52,53]. Together, these models clarify the adsorption mechanism and offer a strong basis for the mechanistic process conditions to understand the adsorption behavior. The adsorption kinetics of Cr(VI) and Hg(II) were evaluated by fitting the experimental *q_t_* data (Figure 5c,d) to the PFO and PSO models through nonlinear regression. The parameters in Table 1 validate the strong reliability of the kinetic models. The PFO model demonstrates a better fit for both Cr(VI) *(R*^2^ = 0.982) and Hg(II) (*R*^2^ = 0.956) than the PSO model (*R*^2^ = 0.968 for Cr(VI) and 0.926 for Hg(II)), supported by its higher *R*^2^ values and minimized error functions. The strong agreement between predicted and measured *q_t_* values confirms the suitability of the PFO model for describing Cr(VI) and Hg(II) adsorption. This supports a removal mechanism based on a physical adsorption process on the CMC-HSDs/Fe_3_O_4_ nanocomposite. Moreover, the low PFO rate constants (*K*_1_ = 0.0225 min^−1^ for Cr(VI); 0.0492 min^−1^ for Hg(II)) reflect a gradual initial uptake on the biocomposite. Similarly, the small PSO constants (K_2_ = 1.121 × 10^−4^ and 4.047 × 10^−4^ g·mg^−1^·min^−1^, respectively) point to a slow but sustained adsorption process that achieves high efficiency over extended time.

The applicability of the Elovich model (*R*^2^ = 0.952 for Cr(VI) and 0.871 for Hg(II)) points to a heterogeneous adsorption surface, a finding that offers crucial understanding of the adsorption mechanism depicted in Figure 5c,d. The Elovich parameters *α* (related to initial adsorption rate) and *β* (related to adsorption energy) quantitatively capture the effects of surface heterogeneity: for Cr(VI), *α* = 0.02 mg·g^−1^·min^−1^ and *β* = 3.54 g·mg^−1^; for Hg(II), *α* = 0.0324 mg·g^−1^·min^−1^ and *β* = 10.801 g·mg^−1^. The moderate *α* parameters suggest prolonged uptake kinetics for both contaminants. The markedly high *β* value indicates a rapid decline in the adsorption rate with increasing surface coverage, which may be associated with increasing energetic barriers, suggesting chemisorption on a heterogeneous surface. This chemisorption mechanism is probably a factor behind the composite’s efficacy in heavy-metal removal for advanced wastewater treatment. Understanding these kinetic phases is operationally valuable, especially under variable contaminant concentrations, as it provides a theoretical framework for predicting adsorption behavior. The application of the Elovich model to the Cr(VI) and Hg(II) adsorption data further elucidates the mechanistic details of the interaction between CMC-HSDs/Fe_3_O_4_ and the target pollutants. Consequently, this approach provides critical insight into adsorption processes while suggesting the remediation protocols for metal-bearing wastewater, thereby reinforcing its utility in environmental engineering contexts [53].

### 3.4. Cr(VI) and Hg(II) Diffusion Mechanism

The linearized intra-particle diffusion (LIPD) model was used to understand the mass transport mechanism for Cr(VI) and Hg(II) adsorption onto the biocomposite. As shown by the kinetic parameters (*k_p_* and *C*) in Table 1 and the linear regression of *qₜ* versus *t*^0·5^ in Figure 4i, the adsorption process follows a triphasic mechanism. The triphasic nature of the plot reveals that adsorption is controlled by complex interfacial dynamics. In the initial stage, film diffusion dominates, characterized by the rapid movement of ions to the adsorbent’s outer surface. This corresponds to the first linear portion of the plot, which does not pass through the origin. The calculated film diffusion rates for Cr(VI) and Hg(II) onto the CMC-HSDs/Fe_3_O_4_ biocomposite are 17.33 and 27.705 mg/g·min^1^/^2^, respectively. The second linear segment then indicates a shift to slower pore diffusion, as ions permeate the material’s interior meso- and micropores. The confined pore geometry imposes significant resistance, lowering the diffusion rate constants to 11.889 (Cr(VI)) and 7.0265 (Hg(II)) mg/g·min^1^/^2^, a marked reduction from the initial film diffusion stage (17.33 and 27.705 mg/g·min^1^/^2^, respectively). The slowest phase, represented by a final linear segment with rates of 0.7224 mg/g·min^1^/^2^ for Cr(VI) and 0.8612 mg/g·min^1^/^2^ for Hg(II), constitutes the rate-limiting step for ion uptake by the CMC-HSDs/Fe_3_O_4_ biocomposite. The polylinear kinetic profile reveals that adsorption is controlled by both film and intra-particle diffusion. The approach to equilibrium resulted from two factors: saturation of the composite’s binding sites and a diminished concentration gradient that could no longer sustain a significant driving force [52].

### 3.5. Isotherm Studies of Cr(VI) and Hg(II) Adsorption on CMC-HSDs/Fe_3_O_4_

#### Essential Equilibrium Models

The equilibrium adsorption data for Cr(VI) and Hg(II) onto the CMC-HSDs/Fe_3_O_4_ biosorbent are presented in Figure 5, with the corresponding parameters for the classical Langmuir and Freundlich isotherm models provided in Table 2. Initial assessment based solely on the coefficient of determination (*R*^2^) was inconclusive, as both models yielded very similar values. To robustly identify the most appropriate model, the Chi-square (*χ*^2^) statistic was evaluated at each temperature. The combined analysis of *R*^2^ and *χ*^2^ (Table 2) confirms that the Langmuir model provides the superior fit for Cr(VI) adsorption, exhibiting consistently higher R^2^ and lower χ^2^ values across all temperatures. This indicates that Cr(VI) sequestration likely occurred via monolayer adsorption onto homogeneous sites with similar affinity on the biosorbent surface. For Hg(II) removal, the data in Table 2 demonstrate a stronger alignment with the Freundlich model. Consequently, Hg(II) adsorption is best described by a multilayer mechanism, involving heterogeneous surface sites with varied functional groups on the CMC-HSDs/Fe_3_O_4_ biocomposite. The Langmuir model shows a better fit than the Freundlich model at 55 °C (Table 2); however, the Freundlich model remains suitable for explaining the Hg(II) removal process. The maximum adsorption capacities (*qₘₐₓ*) derived from the Langmuir model ranged from 198.27 to 232.99 mg/g for Cr(VI) and from 163.49 to 178.74 mg/g for Hg(II) across the temperature range of 25–55 °C (Table 2). The contrasting trends, where Cr(VI) capacity increased while Hg(II) capacity decreased with rising temperature, imply distinct thermodynamic behaviors. This indicates an endothermic process for Cr(VI) adsorption and an exothermic process for Hg(II) uptake. Furthermore, analysis of the Freundlich heterogeneity parameter (*1*/*nF*) yielded values consistently below unity for both metal ions, confirming favorable adsorption conditions. This result further suggests that the sequestration mechanisms involve complex interactions beyond simple surface attachment, likely reflecting the heterogeneous nature of the CMC-HSDs/Fe_3_O_4_ composite matrix [30,54].

### 3.6. Ionic Calculations for the Adsorption Mechanism

A comparative analysis based on *R*^2^ and *χ*^2^ values (Table S3) indicates that the experimental data for Hg(II) adsorption are best described by the advanced double-layer (ADL) assumption, while the Cr(VI) removal process is more accurately calculated by the advanced monolayer (AML) theory. The strong correspondence between these sophisticated calculations and the experimental data is further corroborated by Figure 5d. To achieve a more profound mechanistic insight, key steric and energetic parameters derived from these theories were evaluated as a function of temperature. The steric analysis included the number of adsorbed ions per active site (*n*), the adsorption site density of the CMC-HSDs/Fe_3_O_4_ composite (*N_M_*), and the theoretical maximum uptake capacity (*Q_sat_*). In parallel, the adsorption energy (Δ*E*) was systematically examined as a critical thermodynamic descriptor of the binding mechanism. Each parameter was rigorously analyzed to construct a comprehensive interpretation of the adsorption behavior [55].

#### 3.6.1. Steric Parameters Investigations

A deeper mechanistic understanding of the adsorption process was achieved by examining the steric parameter *n*, which defines the number of adsorbate ions (Cr(VI) or Hg(II)) bound per active site on the CMC-HSDs/Fe_3_O_4_ surface. This parameter is critical for interpreting the spatial distribution and binding interactions of the ions, while also indicating potential adsorbate aggregation in solution. The value of *n* delineates three distinct adsorption regimes [56]: when *n* < 0.5, a single ion interacts with multiple sites, favoring a horizontal orientation; when 0.5 < *n* < 1, a heterogeneous mix of vertical and horizontal configurations is present; and when *n* ≥ 1, multiple ions bind to a single site, indicating multimolecular adsorption with a predominantly vertical orientation.

Based on the quantitative data in Table S3, Figure S2a,b illustrate the variation in the steric parameter *n* with temperature. The values for both Cr(VI) and Hg(II) adsorption exhibit a clear decrease across the studied temperature range (25–55 °C). Specifically, *n* for Hg(II) declines from 3.16 to 1.37, while for Cr(VI) it decreases from 1.35 to 1.08. Critically, all measured *n* values remain above unity, indicating stable adsorption behavior characterized by a multimolecular mechanism and a predominantly vertical orientation of the adsorbates on the CMC-HSDs/Fe_3_O_4_ surface. The observed high removal efficiency is further attributed to a pre-adsorption aggregation phenomenon of the ions in the aqueous phase as proved by *n* values. Notably, variations in temperature within this experimental window did not significantly alter the fundamental binding geometry or the coordination chemistry between the adsorbates and the functional groups of the nanocomposite material.

An analysis of the adsorption site density parameter *N_M_* (Figure S2a,b) was conducted to provide deeper insight into the binding dynamics of Cr(VI) and Hg(II) onto CMC-HSDs/Fe_3_O_4_. The *N_M_* values for both adsorbates increased progressively with temperature, as quantified in Table S3. For Hg(II), the values rose from 24.11 mg/g to 61.49 mg/g over the 25–55 °C range, while a more pronounced enhancement was observed for Cr(VI), with *N_M_* escalating from 132.21 mg/g to 210.16 mg/g. This temperature-driven increase in *N_M_*, which quantifies the availability of active binding sites, is directly linked to the improved removal efficiency observed at elevated temperatures. The positive correlation between temperature and this steric parameter further substantiates the endothermic character of the adsorption process. This effect is likely due to the thermal activation of the composite surface, which increases both the number of active sites and their accessibility.

To further elucidate the adsorption mechanism, the temperature-dependent variation in the theoretical saturation capacity, *Q_sat_*, was examined (Figure S2a,d). This parameter was derived from the steric parameters using the assumption-specific relations *Q_sat_* = 2⋅*n*⋅*N_M_* for the ADL theory and *Q_sat_* = *n*⋅*N_M_* for the AML theory. The results demonstrate a significant, temperature-driven increase in *Q_sat_* for both adsorbates: for Cr(VI), from 178.48 mg/g to 226.97 mg/g, and for Hg(II), from 152.38 mg/g to 168.48 mg/g over the 25–55 °C range. This pronounced enhancement is characteristic of an endothermic adsorption process, signifying favorable interactions at the biocomposite’s active sites as temperature rises. The observed positive correlation between temperature and the parameters *N_M_* and *Q_sat_* can be attributed to thermally activated processes. Elevated temperatures enhance molecular diffusion, increasing the mobility of Cr(VI) and Hg(II) ions within the porous structure of the CMC-HSDs/Fe_3_O_4_ composite. This facilitates more frequent and effective collisions with the newly created adsorption sites. Concurrently, thermal energy likely promotes the exposure and activation of previously inaccessible binding sites on the adsorbent surface.

#### 3.6.2. Interpretation of Energetic Parameters

The adsorption energy values (ΔE) for Cr(VI) and Hg(II) at various solution temperatures are presented in Figure S2c,d. Both primary interaction energies (Δ*E*_1_, adsorbate-composite) and secondary self-interaction energies (Δ*E*_2_, adsorbate-adsorbate) are positive, confirming the endothermic nature of the overall adsorption process on the CMC-HSDs/Fe_3_O_4_ composite. Thermodynamic analysis reveals that the Δ*E*_1_ values for the Cr(VI)-composite and Hg(II)-composite interfaces are consistently greater than the corresponding Δ*E*_2_ values across all temperatures. This consistent energy differential (ΔE_1_ > ΔE_2_) indicates a stronger thermodynamic driving force for adsorbate binding to the composite surface than for intermolecular self-aggregation in solution.

### 3.7. Thermodynamic Studies of Cr(VI) and Hg(II) Uptake

The spontaneity and inherent energetics of the adsorption process were evaluated through a thermodynamic analysis of the Cr(VI) and Hg(II) interactions with the CMC-HSDs/Fe_3_O_4_ adsorbent. Core thermodynamic parameters, entropy, Gibbs free energy, and internal energy, were quantified by fitting the AML and ADL theories to experimental data acquired across a range of temperatures.

#### 3.7.1. Entropy

**Figure 6:**
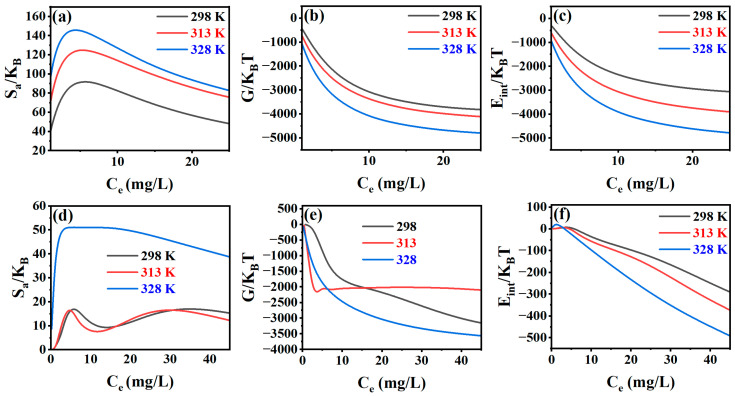
Temperature-dependent evolution of thermodynamic parameters for the adsorption of Cr(VI) (**a**–**c**) and Hg(II) (**d**–**f**): (**a**,**d**) entropy, (**b**,**e**) Gibbs free energy, and (**c**,**f**) internal energy.

Figure 6a,d depict the adsorption entropy for Cr(VI) and Hg(II), respectively, serving as an indicator of disorder at the solid–liquid interface. For Cr(VI), a clear increase in entropy is observed with rising adsorbate concentration up to a maximum value, which occurs prior to half-saturation. This initial trend is attributed to the progressive occupation of available active sites on the CMC-HSDs/Fe_3_O_4_ surface. At this stage, Cr(VI) ions readily access unoccupied sites, resulting in a more disordered system. Beyond half-saturation, the entropy declines, signaling a transition to a more ordered state. This decrease correlates with the diminishing availability of active sites, culminating in zero entropy at complete saturation, where all binding sites are occupied [20]. In contrast, Hg(II) adsorption exhibits a distinct “threshold” behavior, as shown in Figure 6d. The entropy remains relatively low and stable at 298 K and 313 K but undergoes a dramatic increase at 328 K. This sharp thermal dependence suggests that while Cr(VI) adsorption follows a consistent pathway, Hg(II) adsorption undergoes a significant mechanistic shift at elevated temperatures. This shift may result from high-energy processes such as the dehydration of Hg(II) ions or the thermal activation of latent adsorption sites that are inactive at lower temperatures.

#### 3.7.2. Gibbs Free Energy

The evolution of internal energy during the adsorption of Cr(VI) and Hg(II) is presented in Figure 6c,f. This parameter quantifies the energetic stabilization occurring as ions transition from the bulk solution to the adsorbent interface. For Cr(VI) in Figure 6c, the normalized internal energy exhibits a consistent and temperature-dependent decrease, with the steepest descent observed at 328 K. This monotonic reduction signifies a strongly favorable, exergonic stabilization process, where energy is released upon the formation of optimal coordination bonds with the CMC-HSDs/Fe_3_O_4_ surface. Conversely, the profile for Hg(II) in Figure 6f reveals a more complex mechanism. A distinct initial increase, or energy barrier, is evident at low concentrations (particularly at 298 K and 313 K) prior to the eventual energy decrease. Moreover, the Hg(II) isotherms display significantly greater dispersion across the temperature range compared to the closely clustered Cr(VI) data. This pronounced thermal dependence underscores that the internal energy landscape for mercury adsorption is highly sensitive to temperature.

#### 3.7.3. Internal Energy

The variation in internal energy during the adsorption of Cr(VI) and Hg(II) is shown in Figure 6c,f, offering insights into the energetic stability of the system as ions transfer from solution to the adsorbent surface. In Figure 6c, Cr(VI) adsorption exhibits a continuous decrease in internal energy, with a more pronounced decline observed at elevated temperatures. This trend indicates favorable bonding interactions between Cr(VI) ions and the CMC-HSDs/Fe_3_O_4_ surface, as energy is released when ions occupy optimal coordination sites. Conversely, Hg(II) adsorption in Figure 6f displays a distinct energetic profile. A slight initial rise, or “hump,” occurs at very low concentrations (most notably at 298 K and 313 K) before the energy decreases. This suggests an initial energy barrier or activation step is required before Hg(II) ions can stabilize on the surface. Additionally, the internal energy curves for Hg(II) are more widely dispersed across temperatures compared to the closely grouped Cr(VI) data, highlighting the greater sensitivity of the mercury adsorption system to thermal variations and specific interfacial interactions.

### 3.8. Possible Cr(VI) and Hg(II) Adsorption Mechanism

**Figure 7:**
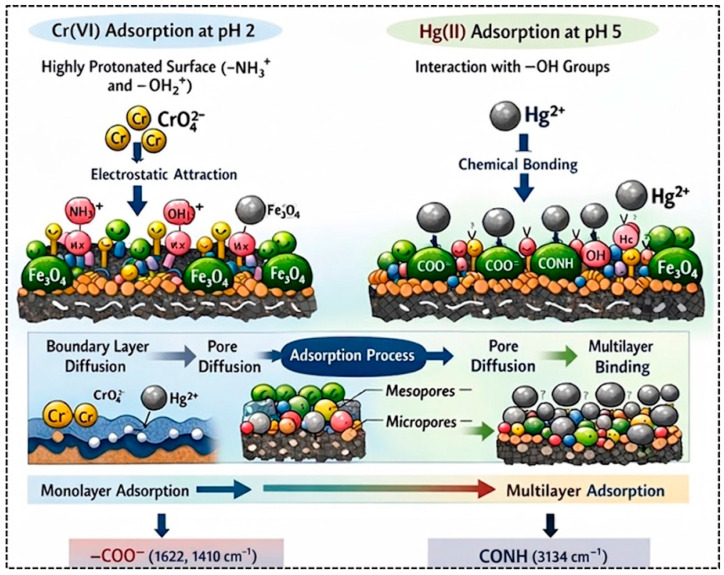
Possible Cr(VI) and Hg(II) adsorption mechanism on CMC-HSDs/Fe_3_O_4_ nanocompos.

The removal of Cr(VI) and Hg(II) by the CMC-HSDs/Fe_3_O_4_ biocomposite operates through a synergistic mechanism involving electrostatic forces, chemical binding, and pore diffusion (Figure 7). At the operational pH of 2, which is well below the material’s pH_pzc_ of 6.67, the surface is strongly protonated. This creates a favorable electrostatic environment for the anionic Cr(VI) species (CrO_4_^2−^) to bind with positively charged –NH_3_^+^ and –OH_2_^+^ sites. In contrast, Hg(II) adsorption at pH 5 proceeds primarily via interaction with surface hydroxyl groups. Spectroscopic analysis identifies the key functional groups responsible for chemisorption: the carboxylate groups of CMC (at 1622 and 1410 cm^−1^) and the amide linkages of HSDs (3134 cm^−1^). Fe_3_O_4_ nanoparticles contribute synergistically by (i) providing Lewis acid sites for coordination with heavy metal ions, (ii) enabling magnetic separation, and (iii) enhancing the structural stability of the biocomposite. The partial positive charge on iron atoms facilitates electrostatic attraction toward anionic Cr(VI) species and promotes surface complexation with Hg(II). The Fe–O bonds (confirmed by FTIR at 609 cm^−1^) act as additional binding centers, where oxygen atoms donate electron density to metal ions, while iron centers coordinate with electron-rich functional groups from HSDs and CMC, creating an interconnected adsorption network [57]. Kinetic data, best described by the LIPD model, indicate a three-stage transport process. Ions initially diffuse through the boundary film, then migrate through the composite’s meso- and micropores, before finally attaining a state of equilibrium saturation.

Thermodynamic assessments confirm the endothermic nature of the adsorption. Both the theoretical saturation capacity (*Q_sat_*) and the density of active sites (*N_M_*) increase at higher temperatures (55 °C), suggesting that thermal energy activates latent binding sites on the adsorbent. Advanced ionic calculations, using the AML and ADL approaches, reveal that the adsorbates bind in a vertical, multimolecular configuration (*n* > 1). This orientation is thermodynamically favored, as the energy of surface adsorption (Δ*E*_1_) exceeds that of lateral adsorbate-adsorbate interaction (Δ*E*_2_). A fundamental difference in the adsorption pathways is observed. Cr(VI) removal follows a straightforward, spontaneous monolayer process. Conversely, Hg(II) adsorption exhibits a more complex, multilayer profile.

### 3.9. Comparison with Other Adsorbents

The performance of the CMC-HSDs/Fe_3_O_4_ composite is compared with recent adsorbents in Table 3, focusing on key operational parameters: optimal solution pH, required contact time, cycle stability, and adsorption capacity for Cr(VI) and Hg(II). The measured adsorption capacities were 236.7 mg/g for Cr(VI) and 162.07 mg/g for Hg(II). These values indicate a performance superior to that of most adsorbents documented in the recent literature. Despite faster kinetics in some materials, CMC-HSDs/Fe_3_O_4_ achieved superior uptake capacities and retained robust performance under diverse conditions. Reusability directly reflects adsorbent stability. Compared to alternative adsorbents, CMC-HSDs/Fe_3_O_4_ showed superior cycle stability, confirming its enhanced durability. Moreover, the superior sustainability of CMC-HSDs/Fe_3_O_4_, coupled with its robust adsorption of Cr(VI) and Hg(II), underscores its strong potential as an ecofriendly biosorbent.

### 3.10. Statistical Optimization Analysis

The statistical significance and reliability of the Cr(VI) and Hg(II) removal model were evaluated using ANOVA. The resulting F-statistics (Table S4), 629.46 and 164.09 respectively, validate the model with high confidence [40]. The high R^2^ values (0.99 for both contaminants) obtained for the BBD model attest to its statistical accuracy and reliability, confirming an excellent fit between the model predictions and the empirical data. The negligible Lack of Fit values (0.0849 for Cr(VI), 0.0026 for Hg(II)) confirm the model’s statistical validity, indicating an accurate fit to the data and strong confidence in the results [68]. Independent variables are considered statistically insignificant if their *p*-value exceeds 0.05. Applying this threshold, the developed removal model identifies the following statistically significant terms for both Cr(VI) and Hg(II) ions: A, B, C, AB, AC, BC, A^2^, B^2^, C^2^. The interdependence of adsorption variables and the resulting response was modeled with a quadratic polynomial expression [69]. The explicit forms of this empirical model for Cr(VI) and Hg(II) are provided in Equations (5) and (6).

Y = +85.36 − 23.63 A + 22.01 B + 23.79 C − 20.95 AB − 18.02 AC + 19.07 − 29 A2 − 29.03 B2 − 25.43 C2(5)

Y = +94.14 − 14.55 A + 15.27 B + 29.59 C − 10.31 AB − 12.21 AC + 11.69 − 32.46 A2 − 19.39 B2 − 27.36 C2(6)

The assessment of model adequacy requires a systematic evaluation of residual patterns. As shown in Figure S3, the residuals exhibit a random distribution around the zero-error line, indicating no systematic bias and confirming that key model assumptions are satisfied. The strong alignment between experimental outcomes and model predictions further validates the model’s accuracy and supports its reliability for predictive use [55]. Additional verification is provided through a direct comparison of predicted versus observed removal efficiencies for both contaminants (Figure S3). The close correspondence between these values reinforces the robustness of the model and affirms the appropriateness of the statistical framework applied [70].

**Figure 8:**
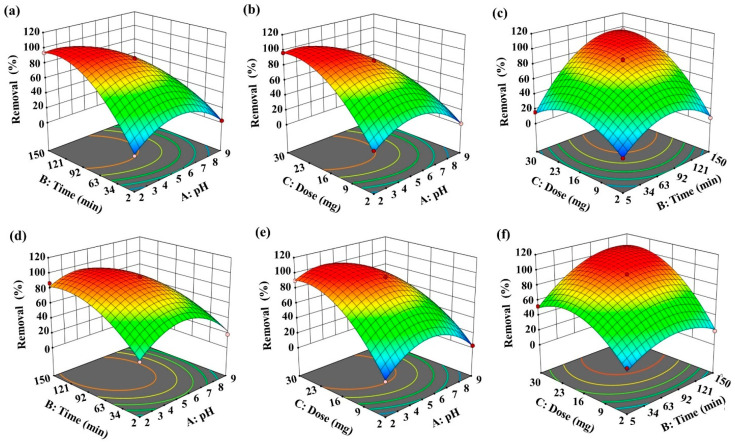
Response surface plots from the BBD for optimizing the adsorption of (**a**–**c**) Cr(VI) and (**d**–**f**) Hg(II) onto CMC-HSDs/Fe_3_O_4_.

To explore the complex interactions among the studied parameters, three-dimensional response surface plots were employed. These visual tools were used to evaluate the combined effects of pH, adsorption time, and CMC-HSDs/Fe_3_O_4_ dosage on the removal efficiency of Cr(VI) and Hg(II). Figure 8a illustrates an inverse correlation between solution pH and Cr(VI) removal, while Figure 8b also shows that Hg(II) degradation is optimal at pH 5. Lowering the pH from 9 to 2 substantially enhanced Cr(VI) elimination. Conversely, Hg(II) removal increased as pH rose from 2 to 5, then decreased at pH values above 5. The pHpzc of CMC-HSDs/Fe_3_O_4_, determined to be 6.67 (Figure S1), further confirms the pivotal role of pH in the removal mechanism. The sequestration efficacy for both Cr(VI) and Hg(II) ions demonstrated a marked increase corresponding to an elevated mass of the CMC-HSDs/Fe_3_O_4_ composite, ranging from 2 to 30 mg per 25 mL solution. This relationship is clearly illustrated in Figure 8b,e. This enhancement is principally ascribed to the greater surface area and augmented density of accessible binding sites concomitant with an increased mass of the adsorbent material. Furthermore, extending the adsorption duration enhances the frequency of interactions between the target ions and the adsorbent surface, thereby improving the sequestration rate for both Cr(VI) and Hg(II) as demonstrated in Figure 8c,f. According to the BBD analysis and subsequent experimental verification, maximum removal of Cr(VI) (93.89%) was attained under the following optimized parameters: a contact period of 150 min, a pH of 2, and an adsorbent quantity of 16 mg. While optimal elimination of Hg(II) (95.08%) was achieved with a contact time of 77.5 min, a pH of 5.5, and an adsorbent dosage of 16 mg.

### 3.11. Adsorption in Complex Environments

#### 3.11.1. Effect of Coexisting Ions

**Figure 9:**
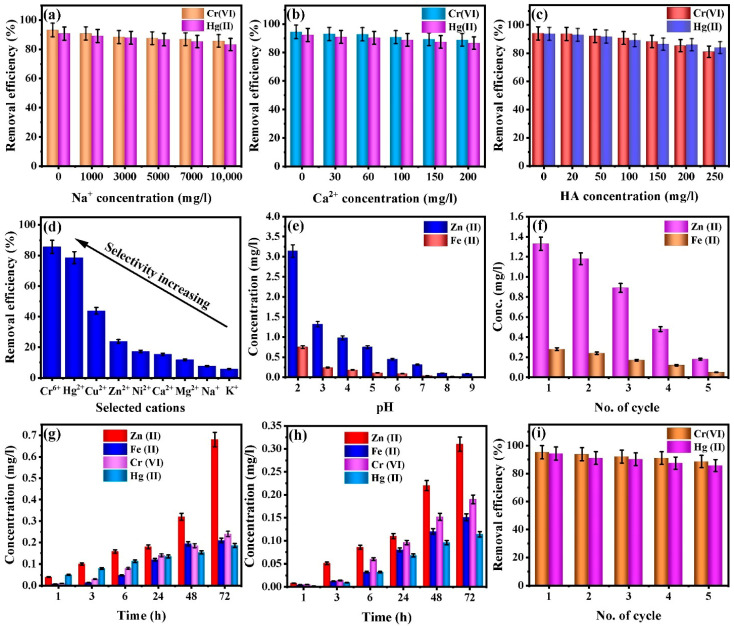
Removal of Cr(VI) and Hg(II) by CMC-HSDs/Fe_3_O_4_: effects of (**a**) Na^+^, (**b**) Ca^2+^, (**c**) humic acid, and (**d**) selectivity; stability and reusability: (**e**) pH influence on Zn(II) and Fe(II) leaching, (**f**) regeneration cycles on Zn(II) and Fe(II) release, (**g**) leaching at pH 4, (**h**) leaching in 0.1 M NaCl, and (**i**) reusability.

Due to the frequent presence of alkali and alkaline-earth metal cations such as sodium (Na^+^) and calcium (Ca^2+^) in real wastewater systems, the extraction efficiency of target ions is often diminished as a result of competitive occupation of available adsorption sites. Given that real-world wastewater contains significant concentrations of competing cations such as Na^+^ and Ca^2+^, this study evaluated their influence on the adsorption of Cr(VI) and Hg(II) by the and CMC-HSDs/Fe_3_O_4_. The concentrations selected for Na^+^ (10,000 mg/L) and Ca^2+^ (200 mg/L) reflect typical levels found in industrial effluent, based on existing literature. Figure 8b and Figure 9a illustrate that the CMC-HSDs/Fe_3_O_4_ biocomposite experienced minimal reduction in adsorption efficacy when exposed to competing ionic species. Specifically, following treatment with solutions containing 10,000 mg/L of sodium ions or 200 mg/L of calcium ions, the material retained removal efficiencies of 85.64% and 88.95% for Cr(VI), and 83.32% and 86.67% for Hg(II), respectively. These results underscore the composite’s pronounced resilience to interference from co-existing ions.

#### 3.11.2. Impact of Dissolved Organic Substances

Building upon these considerations, the prevalence of dissolved organic matter (DOM) in actual wastewater streams necessitates that the fabricated material exhibits robust antifouling properties to ensure consistent and efficient removal of target ionic species [54]. To assess the influence of DOM on adsorption performance, humic acid (HA) was employed as a representative model compound. The impact of its presence on the removal of Cr(VI) and Hg(II) by CMC-HSDs/Fe_3_O_4_ is presented in Figure 9c. Results indicate a marginal decline in Cr(VI) and Hg(II) uptake with increasing humic acid concentration. Notably, even at a concentration of 200 mg/L, the adsorbent retained a high removal efficiency of 81.02% for Cr(VI) and 83.95% for Hg(II). Overall, CMC-HSDs/Fe_3_O_4_ demonstrated a pronounced capacity to effectively capture Cr(VI) and Hg(II) even in the presence of competing ions and potential foulants. This robust resistance to common wastewater interferences substantially enhances the material’s practical viability for real-world water purification applications.

#### 3.11.3. Adsorption Selectivity Toward Different Metals

To characterize the selectivity of the CMC-HSDs/Fe_3_O_4_ adsorbent, its affinity was investigated specifically for cationic species. Following this initial evaluation, the material’s specificity toward Cr(VI) and Hg(II) was examined under competitive conditions involving a complex ionic matrix containing the divalent cations Cu(II), Zn(II), Ni(II), Ca(II), and Mg(II), as well as the monovalent ions Na(I) and K(I). In the competitive adsorption experiments, aqueous solutions containing a uniform concentration (100 mg/L) of each metal ion were contacted with the adsorbent. Analysis of the equilibrium adsorption capacities (Figure 9d) revealed a pronounced selectivity trend for the material, which was established in the following order: Cr(VI) > Hg(II) > Cu(II) > Zn(II) > Ni(II) > Ca(II) > Mg(II) > Na(I) > K(I).

### 3.12. Evaluating Ecosystem Regeneration and Long-Term Stability

To be considered environmentally viable, an adsorbent must possess not only a high adsorption capacity but also a minimal risk of subsequent pollutant release, structural degradation, or component leaching. For this reason, studies focusing on leaching potential and desorption characteristics were conducted. An investigation into the possible leaching of zinc and iron from the CMC-HSDs/Fe_3_O_4_ composite was conducted. This assessment of its environmental safety is a critical parameter for determining practical applicability. To evaluate its environmental stability, a batch leaching study was executed based on an adapted standard methodology [54]. This investigation spanned a pH range from 2 to 10 and included samples that had undergone five regeneration cycles. Analysis of zinc and iron leaching behavior (Figure 9e) indicates a pronounced dependence on pH. The fresh adsorbent demonstrated exceptional stability by limiting the concentrations of leached zinc and iron to a maximum of 1.32 mg/L and 0.24 mg/L, respectively, under the highly acidic condition of pH 3. These results comply with both the WHO standard [71] and the Egyptian guidelines [72] for drinking water. Consistent with theoretical expectations, the leaching of both zinc and iron was enhanced under highly acidic conditions (pH < 3), a phenomenon driven by proton-induced dissolution mechanisms.

The potential for constituent loss was critically assessed through repeated adsorption-regeneration sequences to replicate extended operational conditions. As demonstrated in Figure 9f, the concentrations of leached zinc and iron ions exhibit no statistically significant escalation with successive cycles. For the cycled samples, the concentrations of leached zinc and iron remained within the same minimal range as those observed for the fresh material. The environmental relevance of this data is highlighted by comparing these measured leaching levels against existing regulatory thresholds. All measured concentrations fell well below the relevant regulatory limits for drinking water; specifically, they were substantially lower than both the WHO standard (3 mg/L for zinc) and the Egyptian guideline (0.3 mg/L for iron) [71,72]. These findings suggest that the synthesized CMC-HSDs/Fe_3_O_4_ composite possesses robust structural stability and a low environmental risk profile, confirming its suitability for safe application in water treatment.

To assess the long-term stability of the material for potential disposal through burial or landfilling, a leaching evaluation was conducted. In a simulated mildly acidic environment (pH 4) over 72 h, the cumulative release of sequestered Zn(II), Fe(II), Cr(VI), and Hg(II) remained below 1 mg/L (Figure 9g). Correspondingly, exposure to a high-ionic-strength medium (0.1 M NaCl) yielded a negligible release of these ions, with measured concentrations under 0.5 mg/L (Figure 9h). These results demonstrate minimal remobilization of contaminants under conditions representative of natural aqueous systems, indicating robust and durable sequestration. Such stability is essential for practical remediation applications, where variations in environmental pH and salinity are common.

A desorption test conducted with a 0.1 M HCl solution achieved elution efficiencies of 97.89% for Cr(VI) and 95.47% for Hg(II) during the first cycle. These results confirm that the binding of Cr(VI) and Hg(II) to the adsorbent is largely reversible and governed by pH-dependent mechanisms, such as ion exchange and electrostatic attraction. The composite adsorbent also exhibits superior regenerative capability, maintaining 95.16%, 93.80%, 92.14%, 91.02%, and 88.60% of its initial Cr(VI) uptake efficiency and 94.28%, 91.06%, 90.23%, 87.44%, and 85.69% of its Hg(II) uptake efficiency over five consecutive adsorption–desorption cycles (Figure 9i). This sustained performance verifies the material’s structural integrity and supports its viability for repeated, cost-effective deployment in practical remediation scenarios.

### 3.13. Cost Analysis

Translating a material from laboratory research to industrial-scale water treatment processes depends critically on its economic profile. An evaluation of constituent materials, energy expenditures, and manufacturing logistics indicates that the fabricated composite is economically promising, with a projected unit cost of $25.56/Kg (Table S5). The composite maintains its structural integrity across repeated regeneration phases, decreasing its long-term operational expense to an estimated $5.11/Kg. This substantial reduction markedly improves its economic viability for field application. Consequently, the material achieves an ideal equilibrium between superior contaminant removal efficiency and enduring financial feasibility. The cost-effectiveness of manufacturing processes at production volumes hinges critically on the initial selection of raw materials. This work details a synthetic strategy designed around affordable and environmentally sustainable inputs, specifically the confluence of a renewable biopolymer (cellulose), reclaimed animal processing residues, and engineered magnetic nanoparticles that allow for facile magnetic separation. The outcome of this study is a novel biocomposite that offers both environmental safety and economic viability for application in heavy metal removal.

### 3.14. Design and Implementation of a Scaled Batch Process

In industrial practice, the development of adsorption processes for large-scale applications depends largely on correlations empirically established via batch experimentation. Although data from continuous-flow operations provide a more direct scaling pathway, extensive research confirms that batch-derived parameters remain valuable for initial conceptual and process design. The potential for large-scale implementation of Cr(VI) and Hg(II) ion removal was assessed by scaling up the adsorption performance observed under controlled laboratory conditions for a composite of carboxymethyl cellulose, animal-derived fiber, and magnetic nanoparticles. An assessment of the equilibrium data determined the Langmuir isotherm model. The derived constants from this model were subsequently integrated into a mass balance calculation to formulate the predictive scaling correlation expressed in Equation (7) [30,73].

(7) $$\frac{m}{V}=\frac{\left(C_{0}-C_{e})(1+K_{L}C_{e}\right)}{C_{e}q_{max}\;K_{L}}$$

The equation utilizes two principal operational variables: biosorbent mass, denoted as *m* (g), for the CMC-HSDs/Fe_3_O_4_ composite and the aqueous volume, *V* (L), containing either Cr(VI) or Hg(II) ions. Its theoretical foundation rests upon the differential between the initial (*C*_0_) and equilibrium (*Cₑ*) solute concentrations, both expressed in mg/L. Furthermore, the model integrates the Langmuir isotherm’s fundamental parameters: the Langmuir constant *(K_L_*), which reflects the binding energy of adsorption, and the maximum monolayer uptake (*q_max_*), signifying the theoretical saturation limit of the adsorbent surface.

To evaluate scale-up viability, the composite mass necessary to attain high removal efficiencies (85–95%) for either Cr(VI) or Hg(II) ions was determined across a range of effluent volumes (50–100 L) by application of Eq. (10). These calculations assumed consistent operational parameters—initial concentration (*C*_0_): 100 mg/L, temperature: 25 °C, pH: 3.0 for Cr(VI) and pH: 5.0 for Hg(II) and contact duration: 90 min. The resultant mass values are systematically presented in Figure S4. The long-term cost-effectiveness of the proposed method is substantiated by the robust reusability of the adsorbent material. Its functional integrity and sustained removal efficiency, demonstrated through a minimum of five successive adsorption–desorption operations, directly contribute to reduced operational expenditures. Based on this regenerative performance, the amortized cost of application is calculated to be approximately $5.11/Kg for each usage cycle. To evaluate process economics at scale, a treatment operation targeting 95% contaminant sequestration for a 100 L effluent volume requires an initial adsorbent loading of 684.85 g for chromium and 239.17 g for mercury. The implementation of a regeneration protocol substantially lowers operating costs, achieving an expenditure of approximately $3.50 per 100 L for Cr(VI) and $1.22 for Hg(II) while maintaining a post-regeneration removal efficiency of 88.45% for Cr(VI) and 85.69% for Hg(II). These financial estimates are derived from mass requirement calculations performed under controlled conditions, with key operational parameters held constant. The results, which demonstrate both economical synthesis and effective contaminant elimination, strongly suggest the adsorbent’s promising applicability for industrial water remediation.

## 4. Conclusions

This study fabricated a sustainable magnetic ZnO-doped nanosorbent (CMC-HSDs/Fe_3_O_4_) derived from hydrophobic scleroprotein discards. After thorough characterization, the material’s performance as an adsorbent for Cr(VI) and Hg(II) was optimized using a Box–Behnken design. The optimal conditions yielded maximum removal efficiencies of 93.89% for Cr(VI) at 150 min, pH 2, and a 16 mg dose, and 95.08% for Hg(II) at 77.5 min, pH 5.5, and a 16 mg dose. Equilibrium and kinetic studies revealed that adsorption data were best described by the Langmuir isotherm and pseudo-first-order model for Cr(VI) and by the Freundlich isotherm and pseudo-first-order model for Hg(II). Deeper analysis via advanced ionic calculations indicated multimolecular adsorption, with more than one ion bound per active site. This finding points to a multi-layer process characterized by a preferential vertical orientation of the adsorbates, a behavior consistent across the studied temperature range. Thermal activation further enhanced the adsorption capacity of the CMC-HSDs/Fe_3_O_4_ biocomposite by generating additional active sites. Thermodynamic analysis confirmed an endothermic process for both ions, with adsorption energies exceeding 10 kJ/mol. Collectively, these results demonstrate the successful development of an economically viable and environmentally sustainable biocomposite for efficient heavy metal remediation.

## Figures and Tables

**Table 1:** Kinetic analysis of Cr(VI) and Hg(II) adsorption on CMC-HSDs/Fe_3_O_4_.

Models		Equation			Variables		CMC-HSDs/Fe_3_O_4_-Cr(VI)			CMC-HSDs/Fe_3_O_4_-Hg(II)	
PFO		${\;\;q}_{t}=q_{e}\left(1-e^{-k_{1}t}\right)$			*K*_1_		0.0225			0.0492	
					*q_e_*		122.739			111.848	
					*R* ^2^		0.982			0.956	
					*X* ^2^		10.92			67.443	
					*RMSE*		5.78			7.557	
PSO		$q_{t}=\frac{q_{e}^{2}\;\;k_{2}\;t}{q_{e}\;k_{2}\;t+1}$			*K*_2_		1.121 × 10^−4^			4.0471 × 10^−4^	
					*q_e_*		166.04			131.89	
					*R* ^2^		0.968			0.926	
					*X* ^2^		15.67			95.016	
					*RMSE*		7.561			9.793	
Elovich		*q_t_* = $\frac{1}{\beta }\;Ln(\alpha \beta t+1)$			*α*		0.02			0.0324	
					*β*		3.54			10.801	
					*R* ^2^		0.952			0.871	
					*X* ^2^		20.69			129.89	
					*RMSE*		9.214			12.960	
LIPD model											
CMC-HSDs/Fe_3_O_4_-Cr(VI)											
Step 1				Step 2					Step 3		
*K_int_*_,1_	*C*_1_		*R* ^2^	*K_int_*_,2_		*C*_2_		*R* ^2^	*K_int_*_,3_	*C*_3_	*R* ^2^
17.33	−35.1		0.961	11.889		1.23		0.977	0.7224	104.84	0.985
CMC-HSDs/Fe_3_O_4_- Hg(II)											
*K_int_*_,1_	*C*_1_		*R* ^2^	*K_int_*_,2_		*C*_2_		*R* ^2^	*K_int_*_,3_	*C*_3_	*R* ^2^
27.705	−50.501		0.9235	7.0265		46.87		0.778	0.8612	100.85	0.971

**Table 2:** Adsorption isotherm parameters for Cr(VI) and Hg(II) on CMC-HSDs/Fe_3_O_4_.

Isotherm Model	Variables				
Langmuir	*T* (°C)	*q_max_* (mg/g)	*K_L_* (L/mg)	*R* ^2^	*X* ^2^
Cr(VI)	25	198.27	0.145	0.999	0.289
	40	250.07	0.108	0.949	12.097
	55	232.99	0.217	0.999	0.063
Hg(II)	25	178.74	0.050	0.999	0.470
	40	181.76	0.062	0.998	0.875
	55	163.49	0.139	0.999	0.155
Freundlich		*K_F_*	*1*/*n*		
Cr(VI)	25	51.58	0.33	0.995	2.699
	40	25.04	0.63	0.837	38.82
	55	63.34	0.34	0.997	0.898
Hg(II)	25	17.74	0.52	0.999	0.249
	40	21.30	0.48	0.999	0.075
	55	38.49	0.36	0.998	0.903

**Table 3:** Comparative evaluation of Cr(VI) and Hg(II) adsorption for reference materials.

Cr(VI)						Hg(II)					
Materials	pH	Time (min)	Cycle Number	Q (mg/g)	Ref.	Materials	pH	Time (min)	Cycle Number	Q (mg/g)	Ref.
ClAP	3.0	480	5	91.16	[58]	Sugarcane Bagasse	4.0	60	-	35.71	[59]
LBC-Fe_3_O_4_	3.0	240	7	47.62	[60]	Cu_x_S/PDA@Fe_3_O_4_	5.0	24	6	1394.61	[61]
TEPA-Alg beads	2.0	180	5	76.923	[62]	Fe_3_O_4_ nanoparticles	7.0	120	5	218.17	[45]
Fe_3_O_4_@SiO_2_-NH_2_ particles	1.0	300	5	27.2	[63]	FeOOH-Mn_x_O_y_	6.7	720	7	108.54	[47]
ZMBC	9.31	180	-	30.6	[64]	SDS modified BLP	8–9	1440		31.05	[65]
Rice husk activated magnetic biochar	2.0	120	-	9.97	[66]	NH-3	6.0	3 d	-	95.67	[67]
CMC-HSDs/Fe_3_O_4_	2.0	90	5	236.7	This work	CMC-HSDs/Fe_3_O_4_	5.0	90	5	162.07	This work

## Data Availability

All data generated or analyzed during this study are included in this published article.

## Supplementary Materials

The following supporting information can be downloaded at: https://www.mdpi.com/article/10.3390/nano16090521/s1, Figure S1: pHpzc of CMC-HSDs/Fe_3_O_4_ biocomposite; Figure S2: Steric (n, NM, Qsat) and energetic (ΔE) parameters of the AML and ADL theories as a function of temperature for the adsorption of Cr(VI) (a, c) and Hg(II) (b, d) onto CMC-HSDs/Fe_3_O_4_; Figure S3: Model validation diagnostics: (a) and (c) show normal probability plots for Cr(VI) and Hg(II) residuals, respectively; (b) and (d) compare model-predicted values to actual experimental values for Cr(VI) and Hg(II) removal; Figure S4: Mass of CMC-HSDs/Fe_3_O_4_ nanocomposite required to achieve 80–95% removal of Cr(VI) (a) and Hg(II) (b) from contaminated solutions; Table S1: Independent variables and their levels in the Box-Behnken Design; Table S2: The experimental methodology implemented a BBD where the resulting matrix systematically correlates process conditions with Cr(VI) and Hg(II) elimination efficacy; Table S3: Parameters of the AML and ADL theories for Cr(VI) and Hg(II) adsorption; Table S4: ANOVA studies for Cr(VI) and Hg(II)) removal; Table S5: Total cost involved in preparing 1 kg of CMC-HSDs/Fe_3_O_4_. References [74,75,76,77,78,79] are cited in the Supplementary Materials.
