# Supplementary material for: Economic Design of a Novel Magnetic ZnO-Doped Biocomposite: An Integrated Advanced Ionic Theory and Statistical Physics Approach for Cr(VI) and Hg(II) Remediation
Source: Nanomaterials (Basel). 2026 Apr 25;16(9):521. doi: 10.3390/nano16090521 (PMC13165294; doi:10.3390/nano16090521)
Supplement: Supplementary file 1 [file nanomaterials-16-00521-s001.zip › nanomaterials-4269774-supplementary.pdf]

Economic design of a novel magnetic ZnO-doped biocomposite: An integrated advanced ionic theory and statistical physics approach for Cr(VI) and Hg(II) remediation

Supplementary material

3.5.1 Classical modeling of Cr(VI) and Hg(II) removal by CMC-HSDs/Fe_3O_4 biocomposite

To characterize the adsorption equilibrium, the experimental data were fitted to the nonlinear equations for the Langmuir [1] and Freundlich [2] models (Eqs. S1 and S2).

$$q_e = \frac{q_{\max} K_L C_e}{(1+K_L C_e)} \quad \text{(S1)}$$

$$q_e = K_F \, C_e^{1/nF} \quad \text{(S2)}$$

The modeling parameters include the Langmuir constants q_{max}, defining theoretical monolayer capacity (mg/g), and K_L, representing adsorption affinity (L/mg). For the Freundlich isotherm, the capacity constant K_F (mg/g)(L/mg)$^{(1/n)}$) and the exponential term n describe adsorption intensity and surface heterogeneity. Model suitability was determined by comparing the coefficient of determination (R^2) and the Chi-square statistic (χ^2), as derived in Eqs. S3 and S4 [3].

$$R^2 = 1 - \frac{\Sigma(q_{e,\exp} - q_{e,\mathrm{cal}})^2}{\Sigma(q_{e,\exp} - q_{e,\mathrm{mean}})^2} \quad \text{(S3)}$$

$$\chi^2 = \sum \frac{(q_{e,\exp} - q_{e,\mathrm{cal}})^2}{q_{e,\mathrm{cal}}} \quad \text{(S4)}$$

The symbols $q_{e,\exp}$ (mg/g) and $q_{e,\mathrm{cal}}$ (mg/g) (units: mg/g) correspond to the measured and theoretically derived adsorption capacities at equilibrium for Cr(VI) and Hg(II) ions.

3.5.2. Advanced modeling of Cr(VI) and Hg(II) absorption by CMC-HSDs/Fe_3O_4

Employing a suite of advanced adsorption models—monolayer, double layer, and multilayer frameworks—this investigation conducted a systematic analysis of the interface dynamics involving Cr(VI) and Hg(II) with the energetically active sites present on CMC-

$HSDs/Fe_3O_4$. The application of these distinct models revealed unique steric and energetic characteristics, each of which is subjected to comprehensive examination in the discussion that follows:

3.5.2.1. Advanced monolayer (AML) model

The theoretical model posits that the sequestration of Cr(VI) and Hg(II) ions by CMC-$HSDs/Fe_3O_4$ composite proceeds via a mechanism characterized by a homogeneous energy profile, culminating in the establishment of a single-molecular stratum at the adsorbent surface. Eq. S5 offers the corresponding mathematical formalism for this monolayer adsorption phenomenon [4,5].

$$q_e = \frac{nN_M}{1+\left(\frac{C_{1/2}}{C_e}\right)^n} \qquad \text{(S5)}$$

The variable $C_{1/2}$, measured in milligrams per liter, quantifies the half-saturation concentration, a value derived from the accumulated strata of Cr(VI) and Hg(II) on the CMC-$HSDs/Fe_3O_4$composite surface. The parameters n and N_M correspond to the number of ions bound per receptor site and the concentration of available adsorption sites, respectively, both describing the system's steric characteristics.

3.5.2.2. Advanced double-layer (ADL) model

The proposed model establishes a mechanistic explanation for the interfacial behavior of Cr(VI) and Hg(II), characterized by their binding to active sites on the CMC-$HSDs/Fe_3O_4$ composite via the formation of two separate adsorbate layers. These distinct strata adhere to the CMC-$HSDs/Fe_3O_4$ substrate with distinct binding affinities, quantified by the adsorption energy values ΔE_1 and ΔE_2. The energy term ΔE_1 characterizes the interfacial binding between the CMC-$HSDs/Fe_3O_4$ substrate and either Cr(VI) or Hg(II) ions, while ΔE_2 quantifies the associative

interactions occurring between adjacent adsorbed ions of the same species. The numerical values for all parameters within this model were determined through computational analysis of the mathematical formulation provided in Eq S6 [3].

$$q_e = nN_M \frac{\left(\frac{Ce}{c_1}\right)^n + 2\left(\frac{Ce}{c_2}\right)^{2n}}{1+\left(\frac{Ce}{c_1}\right)^n + \left(\frac{Ce}{c_2}\right)^{2n}} \quad \text{(S6)}$$

In this model, the concentration values C_1 and C_2 (expressed in mg/L) quantify the surface density of the adsorbed Cr(VI) and Hg(II) layers, respectively.

3.5.2.3. Advanced multilayer (AMT) model

The statistical formalism indicates that the deposition of Cr(VI) and Hg(II) is regulated by unique adsorption energies, denoted ΔE_1 and ΔE_2 [5]. Within this theoretical construct, the CMC-HSDs/Fe_3O_4 substrate possesses the capacity to adsorb a maximum of $1 + N_l$ distinct ionic layers. Notably, the parameter N_l demonstrates a direct correlation with either Cr(VI)-Cr(VI) or Hg(II)-Hg(II) formed layers, which are supported by the adsorption energy term ΔE_2. Alternatively, the finite adsorption capacity can be represented through the interfacial interactions occurring between Cr(VI) and the CMC-HSDs/Fe_3O_4 composite or between Hg(II) and the same composite, as quantified by ΔE_1. A direct consequence of this framework is that the aggregate count of adsorbed layers for Cr(VI) and Hg(II), designated as N_t, is defined by the sum: $N_t = 1 + N_l$. The complete analytical expression for calculating all relevant steric and energetic variables within the model is detailed in Eq. S7 [3].

$$q_e = nN_M \frac{\left(\frac{Ce}{c_1}\right)^n \left(1-(N_\ell+1)\left(\frac{Ce}{c_2}\right)^{nN_\ell} + N_\ell\left(\frac{Ce}{c_2}\right)^{n(N_\ell+1)}\right)}{\left(1-\left(\frac{Ce}{c_2}\right)^n\right)\left(1-\left(\frac{Ce}{c_2}\right)^n + \left(\frac{Ce}{c_1}\right)^n - \left(\frac{Ce}{c_1}\right)^n\left(\frac{Ce}{c_2}\right)^{nN_\ell}\right)} \quad \text{(S7)}$$

Generally, the multilayer adsorption framework incorporates five distinct configurations (L_1-L_5) as established in reference [4]:

L_1: *n* and N_ℓ are variable - representing complete multilayer adsorption,

L_2: *n* is adjustable and N_ℓ fixed at zero - corresponding to monolayer coverage,

L_3: *n* is changeable and N_ℓ = 1 characterizing bilayer adsorption,

L_4: *n* is configurable and N_ℓ= 2 describing tri-layer formation, and

L_5: *n* = unity and N_ℓ= zero reverting to the classical Langmuir isotherm.

3.5.2.4. Energetic parameters (ΔE)

According to the principles of the advanced theoretical framework, two distinct adsorption energy values (ΔE_1 and ΔE_2) can be determined. These quantities may be derived through the following computational approach [3–5]:

$$C_1 = C_s e^{-\frac{\Delta E_1}{RT}} \quad \text{(S8)}$$

$$C_2 = C_s e^{-\frac{\Delta E_2}{RT}} \quad \text{(S9)}$$

In this context, C_s represents the solubility of either Cr(VI) or Hg(II) within the given solvent. The proposed model indicates that ΔE_1 corresponds to the direct interfacial interaction between Cr(VI) or Hg(II) and the CMC-HSDs/Fe_3O_4 composite surface, whereas ΔE_2 reflects interactions between ionic species. Consequently, ΔE_1 exhibited significantly greater magnitude than ΔE_2 under identical adsorption temperature conditions.

3.5.3. Thermodynamic analyses

3.5.3.1. Entropy

The degree of molecular organization induced by the adsorption of Cr(VI) and Hg(II) onto the CMC-HSDs/Fe_3O_4 surface was quantified through entropy analysis. This assessment was

performed by applying principles of statistical thermodynamics, specifically utilizing the grand potential (*J*) in relation to the total grand canonical partition function (*Zgc*), as defined by the following relationship [3]:

$$J = -k_B T \ \ln Z_{\mathrm{gc}} = -\frac{\partial \ln Z_{\mathrm{gc}}}{\partial \beta} - T\, S_{\mathrm{a}} \tag{S10}$$

in this expression, β is defined as $1/k_BT$, where k_B represents the Boltzmann constant and T is the absolute temperature (in kelvins) of the adsorption system. Consequently, the associated entropy can be determined using the following equation [3]:

$$\frac{S_{\mathrm{a}}}{k_B} = -\beta \frac{\partial \ln Z_{\mathrm{gc}}}{\partial \beta} + \ln Z_{\mathrm{gc}} \tag{S11}$$

Entropy for AML, as a thermodynamic parameter, was measured as informed below [6].

$$\frac{S_{\mathrm{a}}}{k_B} = -\frac{n N_M \left(\frac{Ce}{c_{\frac{1}{2}}}\right)^n \ln\left[\frac{Ce}{c_{\frac{1}{2}}}\right]}{\left(1+\left(\frac{Ce}{c_{\frac{1}{2}}}\right)^n\right)} + N_M \quad \ln\left[1+\left(\frac{Ce}{c_{\frac{1}{2}}}\right)^n\right] \tag{S12}$$

While, according to DLM, the entropy is given by [4]:

$$\frac{S_{\mathrm{a}}}{k_B} = -N_M\left(\frac{\left(\frac{Ce}{C_1}\right)^n \ln\left(\frac{Ce}{C_1}\right)^n + \left(\frac{Ce}{C_2}\right)^{2n} \ln\left(\frac{Ce}{C_2}\right)^{2n}}{1+\left(\frac{Ce}{C_1}\right)^n + \left(\frac{Ce}{C_2}\right)^{2n}} - \ln\left[1+\left(\frac{Ce}{C_1}\right)^n + \left(\frac{Ce}{C_2}\right)^{2n}\right]\right) \tag{S13}$$

3.5.3.2. Gibbs free energy

The spontaneity of the Cr(VI) and Hg(II) adsorption processes was assessed via the Gibbs free energy, a fundamental thermodynamic parameter. Calculated using Eq. S14 of the AML model, the resulting free energy values provide essential insight into the reaction energetics [5].

$$G = \mu\, Q = k_B T \frac{n\, N_M \ln\left[\frac{Ce}{z_{\mathrm{tr}}}\right]}{\left(1+\left(\frac{c_{\frac{1}{2}}}{Ce}\right)^{n}\right)} \tag{S14}$$

where μ signifies the removed ion's chemical potential.

Moreover, the Gibbs free energy was derived using the advanced DLM according to the following equation [4]:

$$\frac{G}{k_B T} = \ln\left[\frac{C_e}{z_{\mathrm{tr}}}\right]\left(n\, N_M \frac{\left(\frac{C_e}{C_1}\right)^{n} + 2\left(\frac{C_e}{C_2}\right)^{2n}}{1+\left(\frac{C_e}{C_1}\right)^{n} + \left(\frac{C_e}{C_2}\right)^{2n}}\right) \tag{S15}$$

here, z_{tr}represents the translational partition function per unit volume, which is calculated using the expression $\left(\frac{2\pi\, m\, k_B T}{h^2}\right)^{3/2}$, where *m* denotes the molecular mass of the solute dye, and *h* is Planck's constant.

3.5.3.3. Internal energy

The internal energy of Cr(VI) and Hg(II) removal was identified as:

$$E_{\mathrm{int}} = -\frac{\partial \ln Z_{\mathrm{gc}}}{\partial \beta} + \frac{\mu}{\beta}\left(\frac{\partial \ln Z_{\mathrm{gc}}}{\partial \mu}\right) \tag{S16}$$

The internal energy of adsorption for Cr(VI) and Hg(II) was derived from the grand canonical partition function. This thermodynamic quantity was determined using the following expression, which is consistent with the formalism of the monolayer statistical physics model [6].

$$\frac{E_{\mathrm{int}}}{k_B T} = -\frac{N_M\left(\frac{Ce}{c_{\frac{1}{2}}}\right)^{n}}{\left(1+\left(\frac{Ce}{c_{\frac{1}{2}}}\right)^{n}\right)}\left(n\ \ln\left[\frac{Ce}{c_{\frac{1}{2}}}\right] -\ \ln\left[\frac{Ce}{z_{\mathrm{tr}}}\right]\right) \tag{S17}$$

The adsorption process for Cr(VI) and Hg(II) was characterized as a dual-energy double-layer system, and its internal energy was determined using the following expression [4]:

$$\frac{E_{\text{int}}}{k_B T} = -N_M \frac{\left(\frac{C_e}{C_1}\right)^n \ln\left(\frac{C_e}{C_1}\right)^n + 2\left(\frac{C_e}{C_2}\right)^{2n} \ln\left(\frac{C_e}{C_2}\right)^{2n} + \frac{1}{\beta}\ln\left(\frac{C_e}{z_{\text{tr}}}\right)\left(\left(\frac{C_e}{C_1}\right)^n + \left(\frac{C_e}{C_2}\right)^{2n}\right)}{1 + \left(\frac{C_e}{C_1}\right)^n + \left(\frac{C_e}{C_2}\right)^{2n}} \quad (S18)$$

Table S1. Independent variables and their levels in the Box-Behnken Design.

Factor	Name	Units	Ranges	Minimum	Mean	Maximum
A	Aqueous pH	-	2-9	2	5.5	9
B	Composite mass	mg	2-30	2	16	30
C	Interaction time	min	5-150	5	77.5	150

Table S2. The experimental methodology implemented a BBD where the resulting matrix systematically correlates process conditions with Cr(VI) and Hg(II) elimination efficacy.

Run order	Variables			Removal (%)			
				Cr(VI)		Hg(II)	
	pH	Time (min0	Dose (mg)	Actual	Predicted	Actual	Predicted
1	2	77.5	2	14.87	12.76	4.98	7.09
2	9	5	16	2.69	2.65	18.27	22.79
3	5.5	5	30	15.69	13.62	52.43	50.02
4	5.5	150	30	95.68	95.78	99.91	103.95
5	5.5	77.5	16	85.72	85.36	94.57	94.14
6	9	150	16	6.97	4.76	34.64	32.71
7	5.5	5	2	4.28	4.18	18.27	14.23
8	5.5	77.5	16	86.97	85.36	93.18	94.14
9	2	5	16	5.81	8.02	29.33	31.27
10	2	77.5	30	96.53	96.39	90.21	90.68
11	5.5	77.5	16	83.69	85.36	95.08	94.14
12	5.5	77.5	16	84.17	85.36	92.98	94.14
13	9	77.5	2	1.39	1.53	2.87	2.4
14	2	150	16	93.89	93.93	86.95	82.44
15	9	77.5	30	10.97	13.08	39.27	37.16
16	5.5	77.5	16	86.27	85.36	94.91	94.14
17	5.5	150	2	7.98	10.05	18.98	21.39

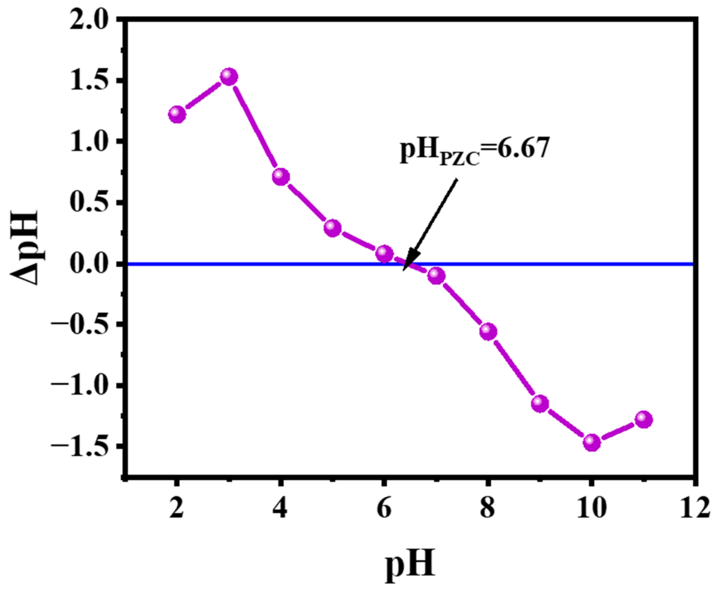

Figure S1. pHpzc of CMC-HSDs/Fe_3O_4 biocomposite.

Table S3. Parameters of the AML and ADL theories for Cr(VI) and Hg(II) adsorption.

Adsorbate	*T (°C)*	*n*	N_M *(mg/g)*	Q_{sat} *(mg/g)*	ΔE_1 *(KJ/mol)*	ΔE_2 *(KJ/mol)*	R^2	X^2
Hg(II)	25	3.16	24.11	152.38	14.06	11.76	0.999	2.372
	40	3.33	23.59	157.11	15.36	12.86	0.999	2.985
	55	1.37	61.49	168.48	17.60	14.60	0.999	0.514
Cr(VI)	25	1.35	132.21	178.48	23.19	0	0.999	0.448
	40	1.13	179.87	203.25	24.51	0	0.999	0.330
	55	1.08	210.16	226.97	26.21	0	0.996	17.46

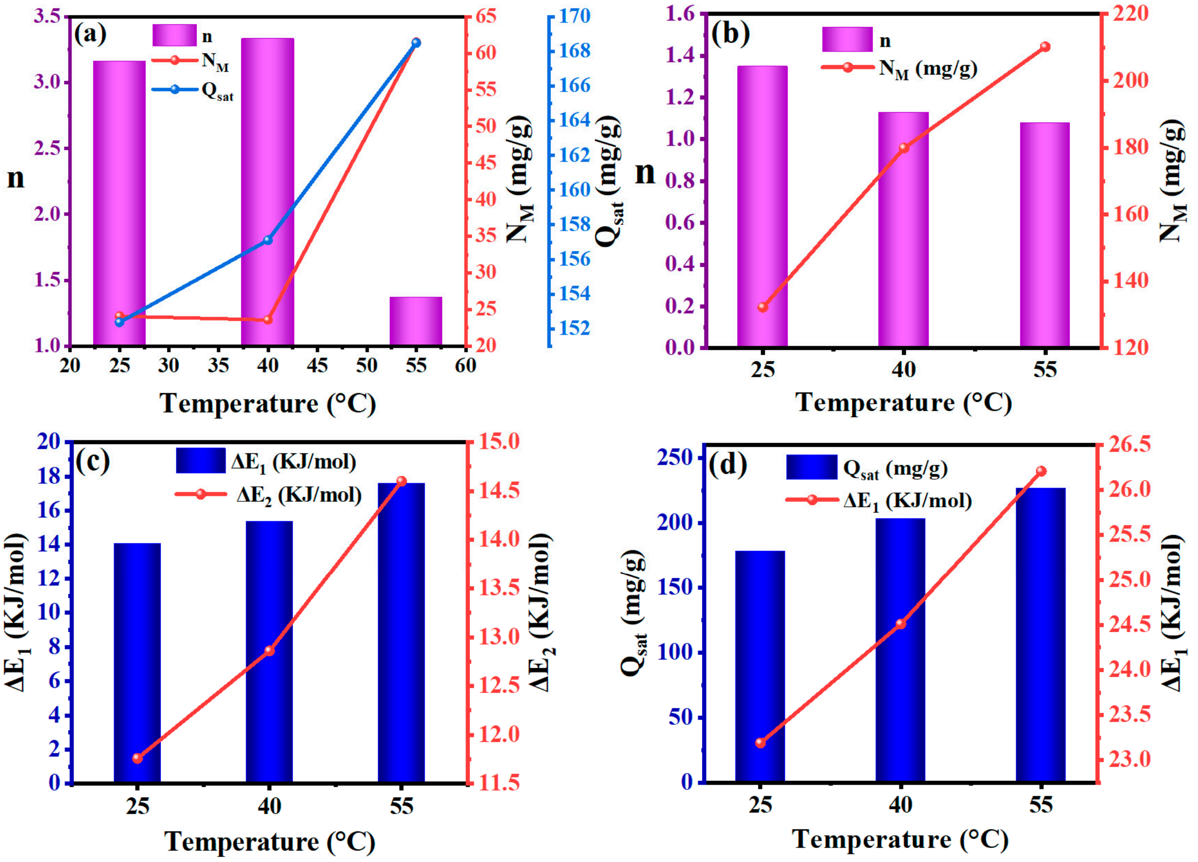

Figure S2. Steric (n, N_M, Q_{sat}) and energetic (ΔE) parameters of the AML and ADL theories as a function of temperature for the adsorption of Cr(VI) (a, c) and Hg(II) (b, d) onto CMC-HSDs/ Fe_3O_4.

Table S4. ANOVA studies for Cr(VI) and Hg(II)) removal.

Source	Cr(VI)					Hg(II)				
	Sum of Squares	df	Mean Square	F-value	p-value	Sum of Squares	df	Mean Square	F-value	p-value
Model	28340.39	9	3148.93	629.46	< 0.0001	22319.94	9	2479.99	164.09	< 0.0001
A-Solution pH	4468.91	1	4468.91	893.32	< 0.0001	1694.2	1	1694.2	112.1	< 0.0001
B-Agitation time	3874.2	1	3874.2	774.44	< 0.0001	1865.99	1	1865.99	123.47	< 0.0001
C-Adsorbent dose	4529.14	1	4529.14	905.36	< 0.0001	7004.54	1	7004.54	463.47	< 0.0001
AB	1755.61	1	1755.61	350.94	< 0.0001	425.39	1	425.39	28.15	0.0011
AC	1298.88	1	1298.88	259.64	< 0.0001	596.09	1	596.09	39.44	0.0004
BC	1455.04	1	1455.04	290.86	< 0.0001	546.86	1	546.86	36.18	0.0005
A^2	3540.01	1	3540.01	707.64	< 0.0001	4435.27	1	4435.27	293.47	< 0.0001
B^2	3547.95	1	3547.95	709.23	< 0.0001	1583.16	1	1583.16	104.75	< 0.0001
C^2	2722.51	1	2722.51	544.22	< 0.0001	3150.89	1	3150.89	208.49	< 0.0001
Residual	35.02	7	5			105.79	7	15.11		
Lack of Fit	27.26	3	9.09	4.69	0.0849	101.86	3	33.95	34.57	0.0026
Pure Error	7.75	4	1.94			3.93		4	0.9821	
Cor Total	28375.41	16				22425.73		16		

Table S5: Total cost involved in preparing 1 kg of CMC-HSDs/Fe_3O_4.

Material	Experimental mass yield (Kg/g)	Total procurement cost (USD)	Cost per acquired unit (kg or g/USD)	Quantity of material utilized (by mass or volume)	Production process material outlay (USD)
HSDs	5 kg	-	-	800 g	-
$ZnCl_2$	500 g	33.48	0.067/g	50 g	3.35
$FeCl_3\cdot 6H_2O$	500 g	9.72	0.02/g	374 g	7.48
$FeSO_4.7H_2O$	500 g	8.39	0.02/g	192 g	3.84
NH_4OH	1 L	3.39	0.003/mL	400 mL	1.356
CMC	200 g	99.90	0.499/g	10 g	4.99
Production Machinery	Time (h)	Certified power output limit (kW/h)	Energy unit cost (USD/kW)	Total cost	
Calcination	2	1	0.28	0.56	
Drying	12	1	0.24	2.88	
Stirrer	2.5	1	0.24	0.60	
		Total yield cost = 25.06 USD For 1.020 kg	Total yield cost 25.56 USD/kg		

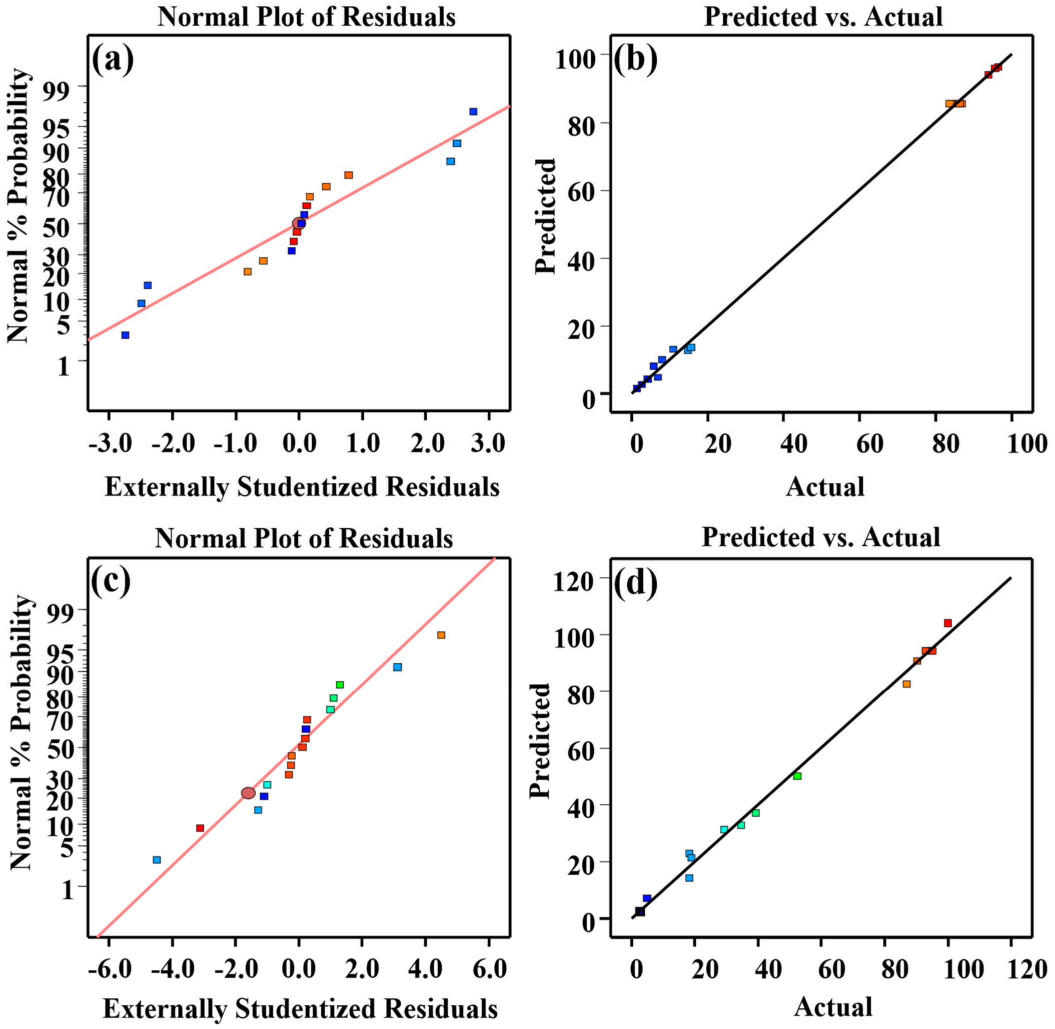

Figure S3. Model validation diagnostics: (a) and (c) show normal probability plots for Cr(VI) and Hg(II) residuals, respectively; (b) and (d) compare model-predicted values to actual experimental values for Cr(VI) and Hg(II) removal.

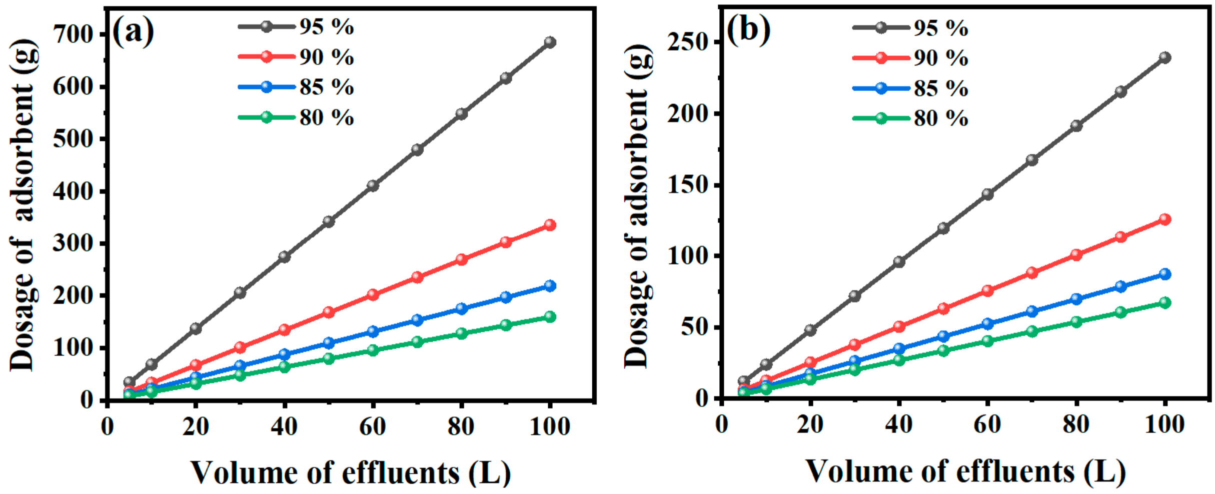

Figure S4.Mass of CMC-HSDs/Fe_3O_4 nanocomposite required to achieve 80–95% removal of Cr(VI) (a) and Hg(II) (b) from contaminated solutions.

References

1. Uliana, A.A.; Bui, N.T.; Kamcev, J.; Taylor, M.K.; Urban, J.J.; Long, J.R. Ion-Capture Electrodialysis Using Multifunctional Adsorptive Membranes. *Science (1979).* 2021, *372*, 296–299, doi:10.1126/science.abf5991.

2. Gupta, K.; Joshi, P.; Gusain, R.; Khatri, O.P. Recent Advances in Adsorptive Removal of Heavy Metal and Metalloid Ions by Metal Oxide-Based Nanomaterials. *Coord. Chem. Rev.* 2021, *445*, 214100, doi:10.1016/j.ccr.2021.214100.

3. Mobarak, M.; Mohamed, E.A.; Selim, A.Q.; Mohamed, F.M.; Sellaoui, L.; Bonilla-Petriciolet, A.; Seliem, M.K. Statistical Physics Modeling and Interpretation of Methyl Orange Adsorption on High–Order Mesoporous Composite of MCM–48 Silica with Treated Rice Husk. *J. Mol. Liq.* 2019, *285*, 678–687, doi:10.1016/j.molliq.2019.04.116.

4. Sellaoui, L.; Sghaier, N.; Erto, A. Outstanding Adsorption of Reactive Red 2 and Reactive Blue 19 Dyes on MIL-101 (Cr): Novel Physicochemical Analysis of Underlying

Mechanism Through Statistical Physics Modeling. *Water 2025, Vol. 17,* 2025, *17*, doi:10.3390/w17111665.

5. Elshimy, A.S.; Abdel-Gawwad, H.A.; Sharib, A.A.A.A.; EL-Gawaad, N.S.A.; Al-Ahmed, Z.A.M.; Bonilla-Petriciolet, A.; Li, Z.; Mobarak, M.; Selim, A.Q.; Seliem, M.K. A New Alkali-Activated Binder Prepared from Dolomite Waste and Diatom Frustules: Insights into the Mechanical Performance and Mn(VII) Treatment. *J. Environ. Chem. Eng.* 2023, *11*, 110392, doi:10.1016/j.jece.2023.110392.

6. Yacoub, S.I.; Saber, S.G.; Ali, R.A.M.; Lima, E.C.; dos Reis, G.S.; Al-Olayan, E.; Salama, Y.F.; Mobarak, M.; Seliem, M.K. CTAB-Modified Alkali-Activated Binder Derived from Favia Corals and Glass Waste: A Novel Bio-Based Adsorbent for Effective Removal of Mn(VII) Ions from Aqueous Solutions. *Journal of Industrial and Engineering Chemistry* 2025, *147*, 406–421, doi:10.1016/j.jiec.2024.12.031.
